# Supplementary material for: One mean to rule them all? The arithmetic mean based egg reduction rate can be misleading when estimating anthelminthic drug efficacy in clinical trials
Source: PLoS Negl Trop Dis. 2020 Apr 8;14(4):e0008185. doi: 10.1371/journal.pntd.0008185 (PMC7170292; doi:10.1371/journal.pntd.0008185)
Supplement: S1 File — (PDF) [file pntd.0008185.s001.pdf]

Survey: geometric vs. Arithmetic mean for helminth egg burden estimation

We would like to deeply thank you for your participation.

In helminthology, the “egg per gram of faeces (EPG)” is used as indicator for the severity of an infection. To quantify the effect of an intervention (e.g. anthelmintic drug), two main measures are usually applied: the Cure Rate (CR) and the Egg Reduction Rate (ERR). The CR depends highly on the baseline infection intensity; therefore, the ERR is more suitable for comparisons among different settings. However, the ERR can be calculated in different ways; whereby the arithmetic and geometric mean approaches are the currently exclusively used. The two approaches are characterised by different strength and weaknesses, e.g. the arithmetic mean is greatly influenced by outliers and skewed distributions while the geometric mean lacks an intuitive interpretation in the context of egg counts. Several examples from the scientific literature show, that the two approaches may result in substantially different estimates of the ERR.
The main objective of this survey is to answer the question: "Which measure of central tendency is most suitable to describe worm burden and treatment efficacy in terms of egg reduction rates?” In detail, we will calculate a series of different measures of ERR (e.g. arithmetic/geometric means, quantiles, applying truncation or Winsorisation, etc.) to identify the approach which is in best agreement with the opinions of experts.

Below, you find samples of random egg counts from different parasites in human stool samples. All data originate from randomized clinical drug trials.

- Always 2 treatment arms are shown (A-red/B-blue)
- The upper row (graph 1-3) shows eggs per gram. For some species and distribution of the log transformed data might be easier to interpret (especially after treatment), they are presented in the lower row.
- The box-plots present the min, Q1, median, Q3, max, dots represent outliers. The treatment effect is in fact the change from baseline to follow-up.

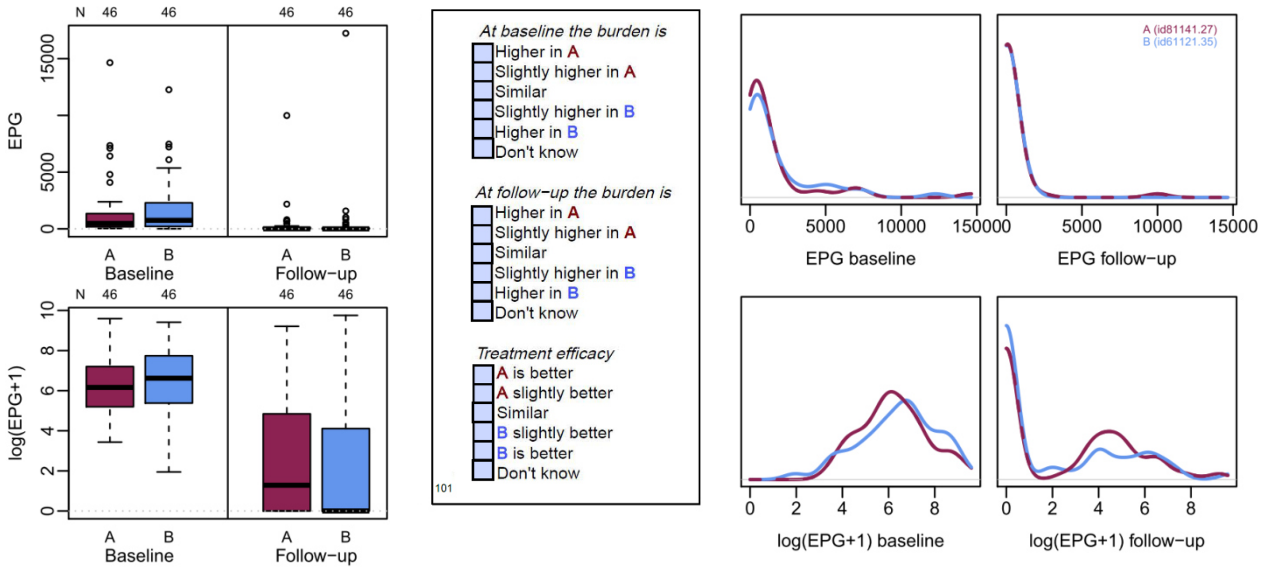

In case the box-plots are not sufficient for a judgement, the line graphs on the right present the density distributions. The density can be interpreted in the same way as a histogram, except that instead of bars a continuous line is shown (to avoid over plotting, see example right). At baseline EPG can be small but not zero; however, at follow-up the value associated with 0 EPG represents the CR.

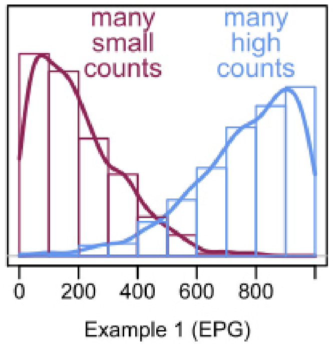

Answer the questions:

1. Please look at the box plots and answer which treatment arm has in your opinion the higher burden at baseline and follow-up and which treatment performed better.
2. If the box plots are unclear, the graphs on the right provide additional information.

Please return the form to: Swiss TPH, Wendelin Moser, wendelin.moser@unibas.ch If you have any questions, don't hesitate to ask me, Tel: +41 61 284 8486.

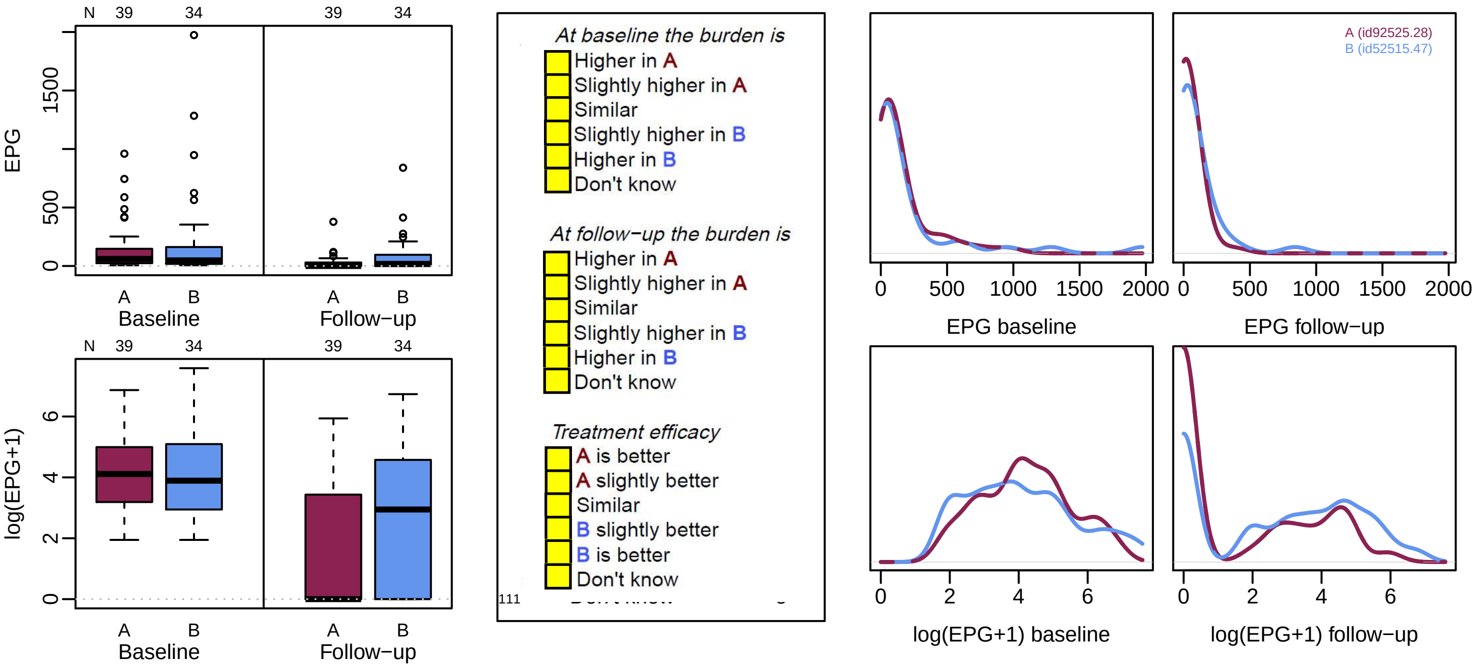
N 39 34 39 34
EPG
log(EPG+1)
Baseline
Follow−up
At baseline the burden is
Higher in A
Slightly higher in A
Similar
Slightly higher in B
Higher in B
Don't know
At follow−up the burden is
Higher in A
Slightly higher in A
Similar
Slightly higher in B
Higher in B
Don't know
Treatment efficacy
A is better
A slightly better
Similar
B slightly better
B is better
Don't know
111
A (id92525.28)
B (id52515.47)
EPG baseline
EPG follow−up
log(EPG+1) baseline
log(EPG+1) follow−up

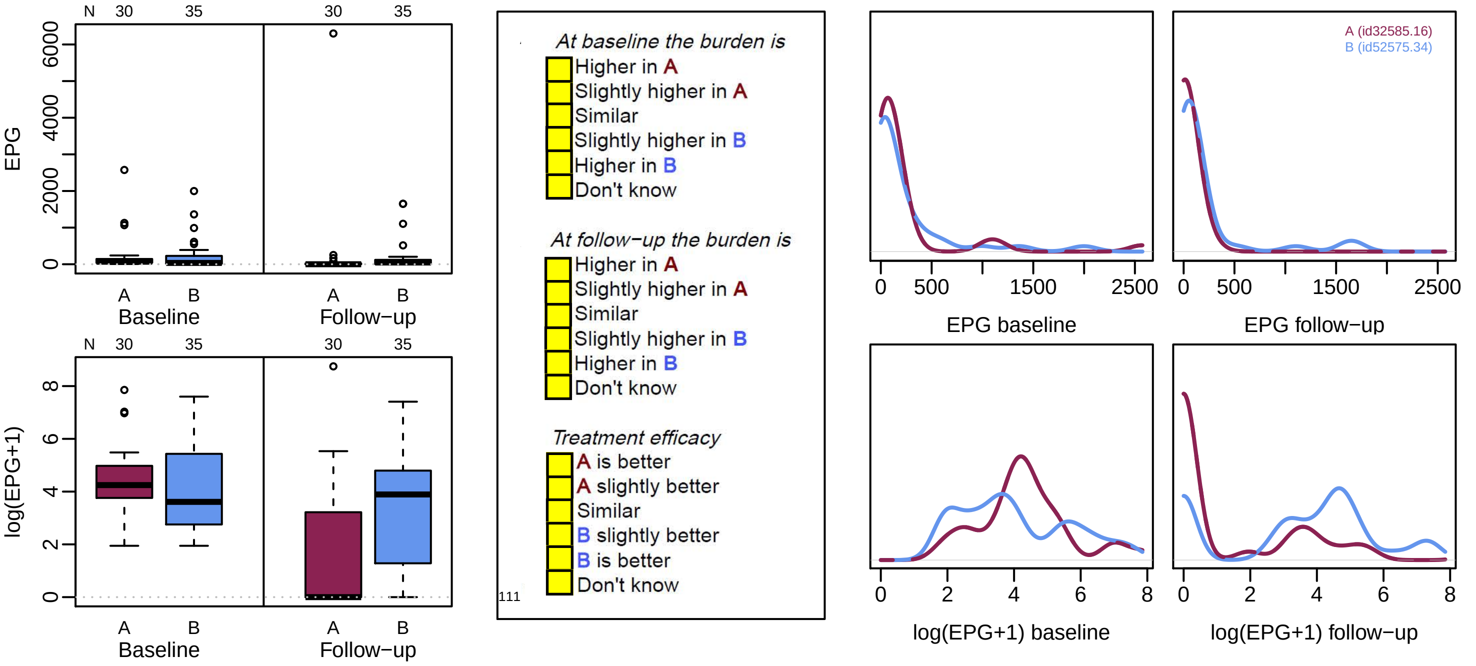
N 30 35 30 35
EPG
log(EPG+1)
Baseline
Follow−up
At baseline the burden is
Higher in A
Slightly higher in A
Similar
Slightly higher in B
Higher in B
Don't know
At follow−up the burden is
Higher in A
Slightly higher in A
Similar
Slightly higher in B
Higher in B
Don't know
Treatment efficacy
A is better
A slightly better
Similar
B slightly better
B is better
Don't know
111
A (id32585.16)
B (id52575.34)
EPG baseline
EPG follow−up
log(EPG+1) baseline
log(EPG+1) follow−up

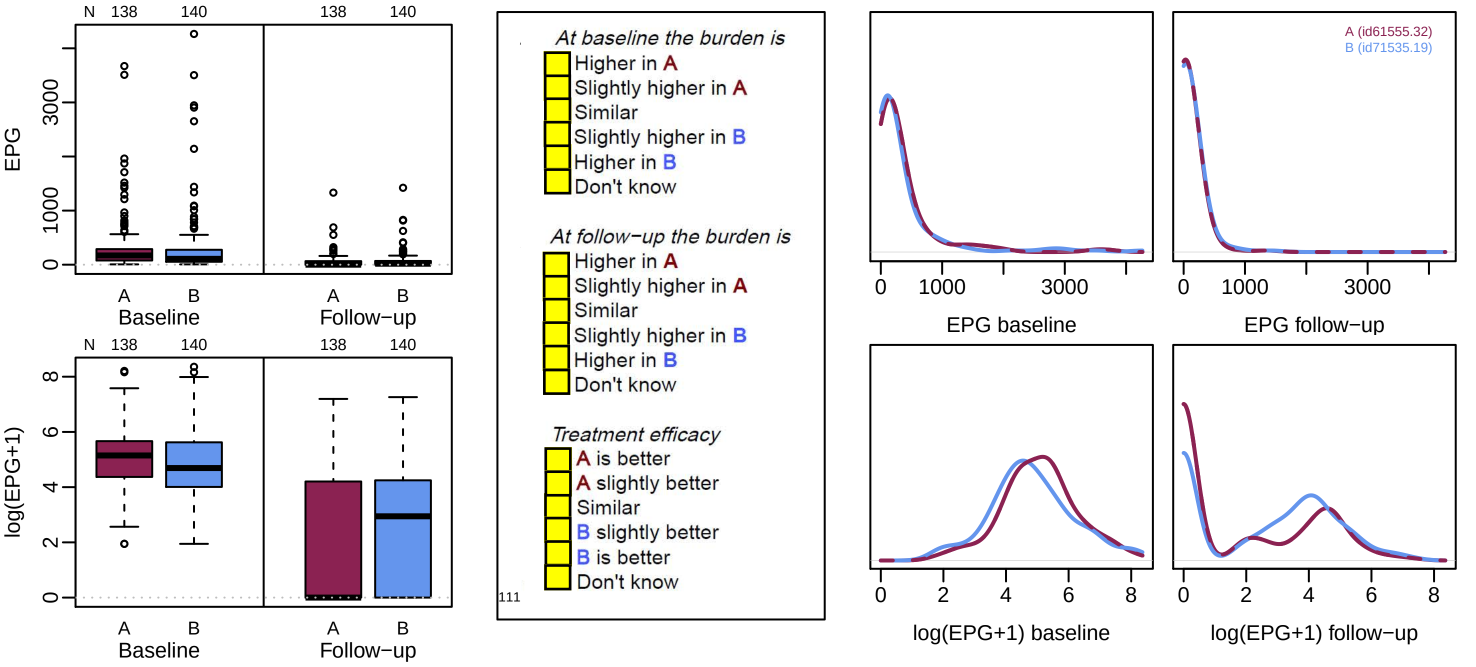
N 138 140 138 140
EPG
log(EPG+1)
Baseline
Follow−up
At baseline the burden is
Higher in A
Slightly higher in A
Similar
Slightly higher in B
Higher in B
Don't know
At follow−up the burden is
Higher in A
Slightly higher in A
Similar
Slightly higher in B
Higher in B
Don't know
Treatment efficacy
A is better
A slightly better
Similar
B slightly better
B is better
Don't know
111
A (id61555.32)
B (id71535.19)
EPG baseline
EPG follow−up
log(EPG+1) baseline
log(EPG+1) follow−up

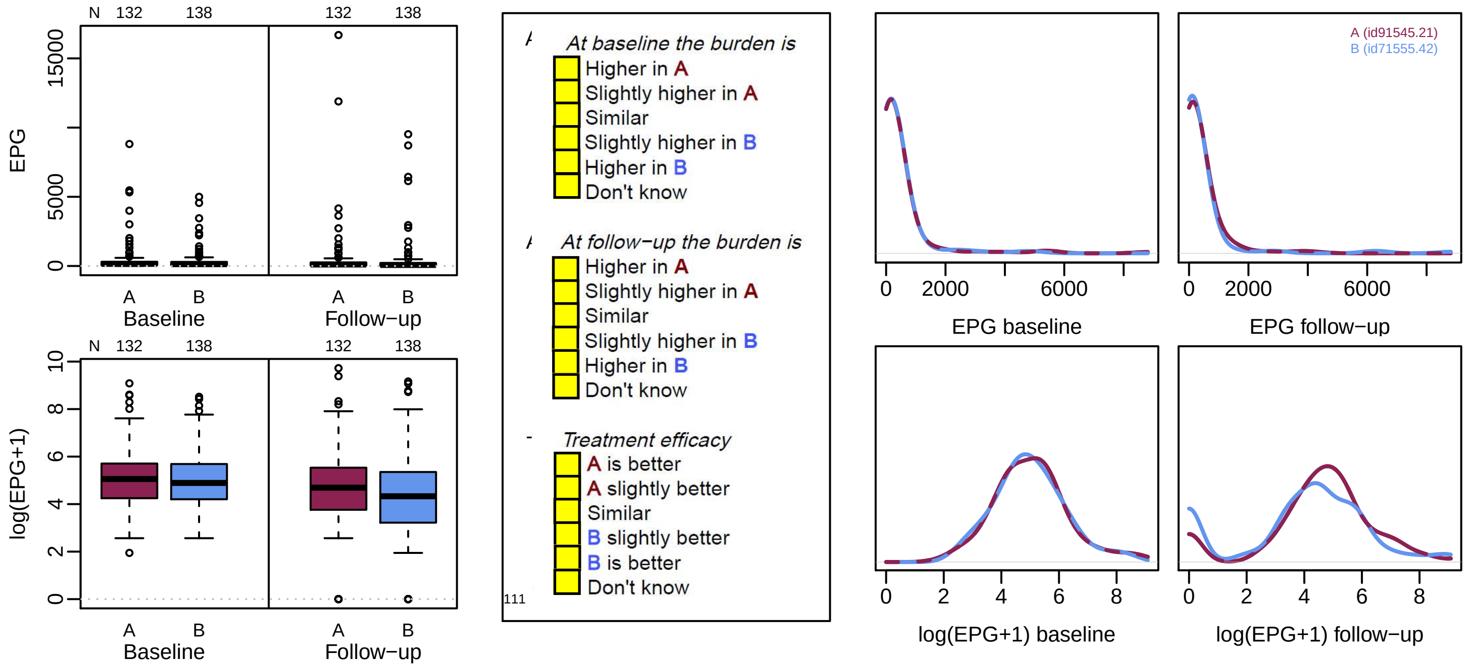
N 132 138 132 138
EPG
log(EPG+1)
A B
Baseline
Follow−up
At baseline the burden is
Higher in A
Slightly higher in A
Similar
Slightly higher in B
Higher in B
Don't know
At follow−up the burden is
Higher in A
Slightly higher in A
Similar
Slightly higher in B
Higher in B
Don't know
Treatment efficacy
A is better
A slightly better
Similar
B slightly better
B is better
Don't know
111
A (id91545.21)
B (id71555.42)
EPG baseline
EPG follow−up
log(EPG+1) baseline
log(EPG+1) follow−up

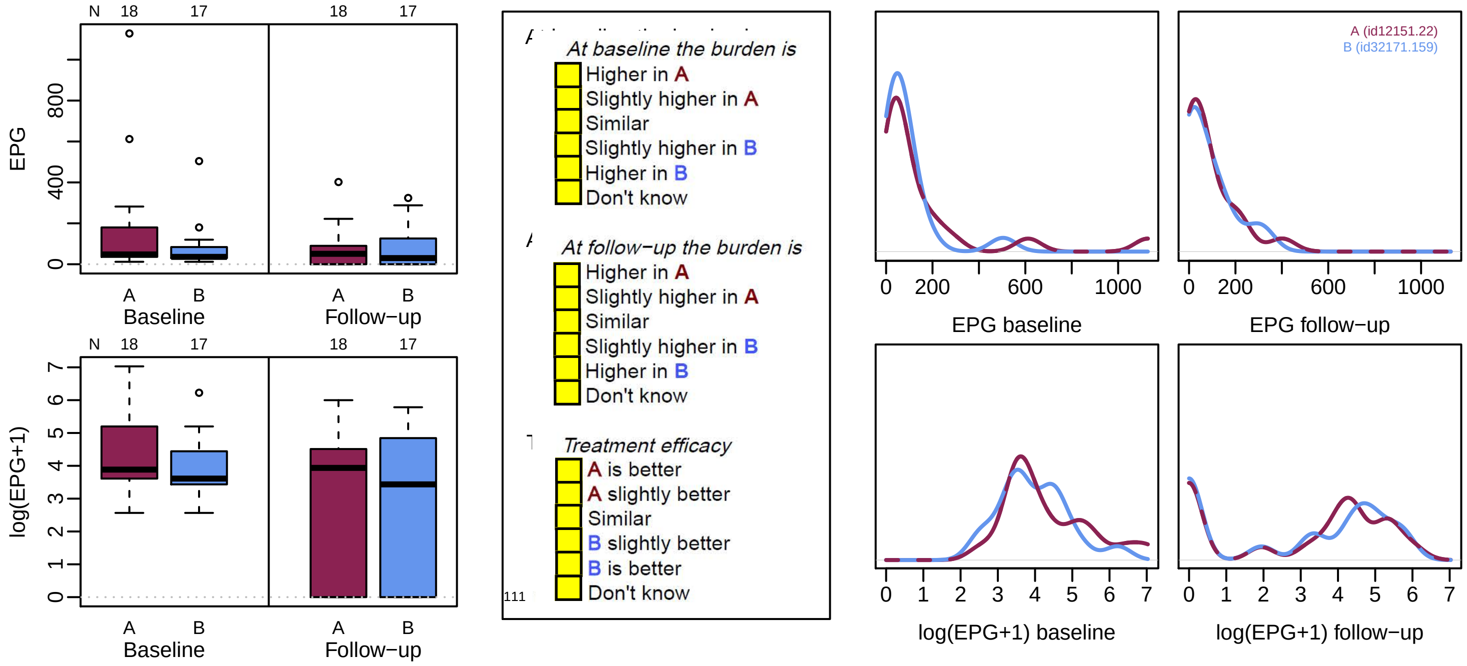
N 18 17 18 17
EPG
log(EPG+1)
A B
Baseline
Follow−up
At baseline the burden is
Higher in A
Slightly higher in A
Similar
Slightly higher in B
Higher in B
Don't know
At follow−up the burden is
Higher in A
Slightly higher in A
Similar
Slightly higher in B
Higher in B
Don't know
Treatment efficacy
A is better
A slightly better
Similar
B slightly better
B is better
Don't know
111
A (id12151.22)
B (id32171.159)
EPG baseline
EPG follow−up
log(EPG+1) baseline
log(EPG+1) follow−up

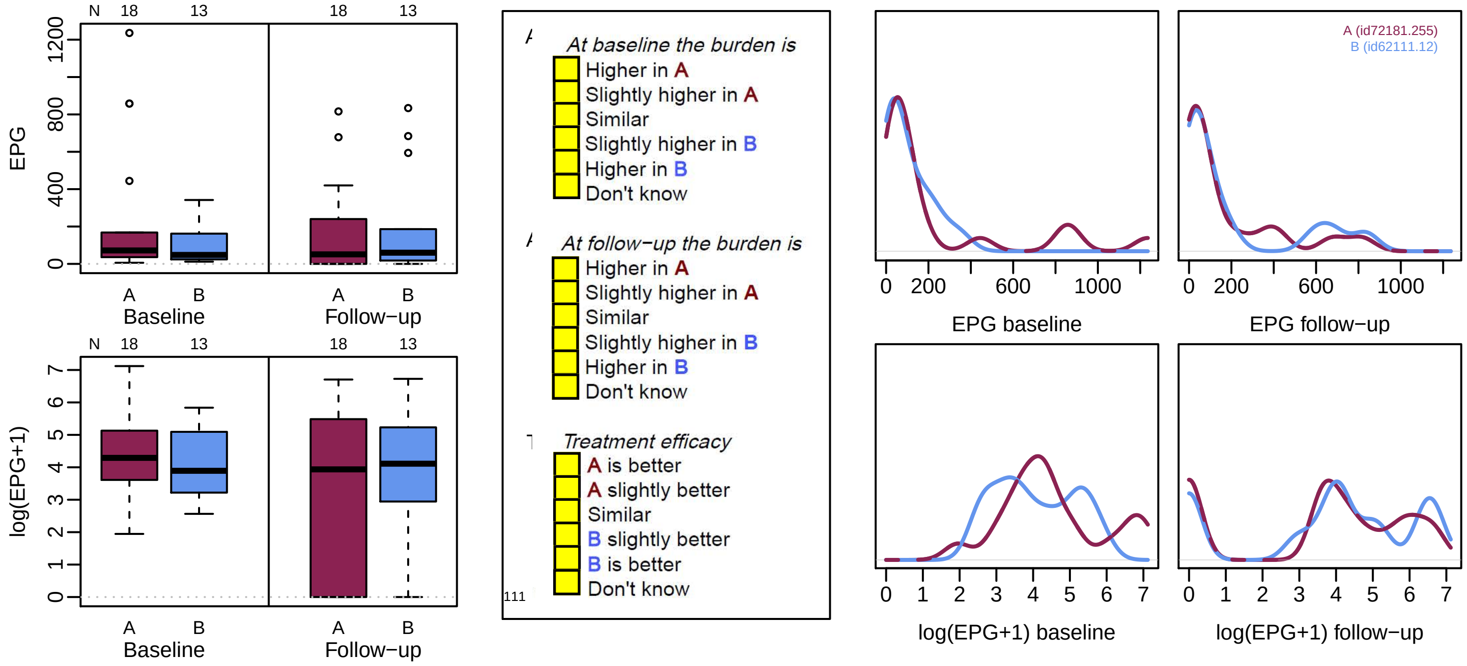
N 18 13 18 13
EPG
log(EPG+1)
A B
Baseline
Follow−up
At baseline the burden is
Higher in A
Slightly higher in A
Similar
Slightly higher in B
Higher in B
Don't know
At follow−up the burden is
Higher in A
Slightly higher in A
Similar
Slightly higher in B
Higher in B
Don't know
Treatment efficacy
A is better
A slightly better
Similar
B slightly better
B is better
Don't know
111
A (id72181.255)
B (id62111.12)
EPG baseline
EPG follow−up
log(EPG+1) baseline
log(EPG+1) follow−up

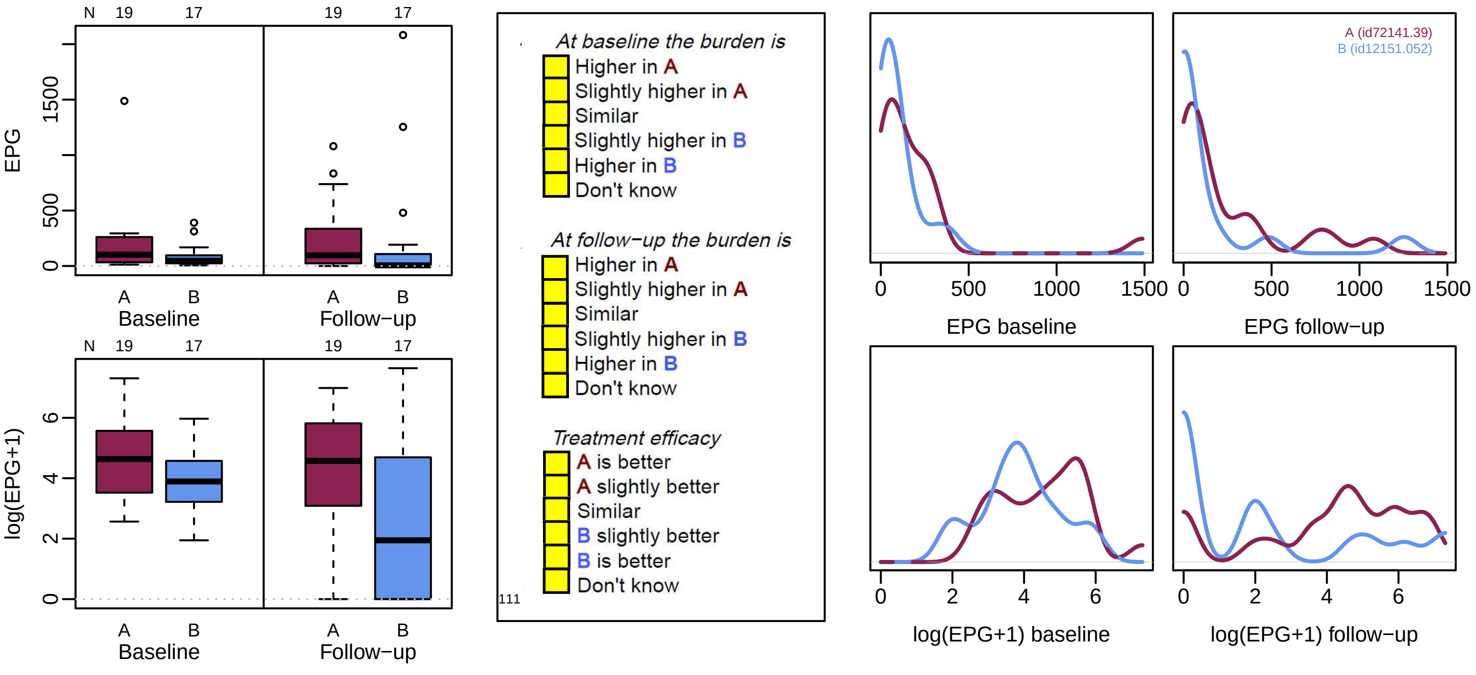
N 19 17 19 17
EPG
Baseline
Follow−up
log(EPG+1)
At baseline the burden is
Higher in A
Slightly higher in A
Similar
Slightly higher in B
Higher in B
Don't know
At follow−up the burden is
Higher in A
Slightly higher in A
Similar
Slightly higher in B
Higher in B
Don't know
Treatment efficacy
A is better
A slightly better
Similar
B slightly better
B is better
Don't know
111
A (id72141.39)
B (id12151.052)
EPG baseline
EPG follow−up
log(EPG+1) baseline
log(EPG+1) follow−up

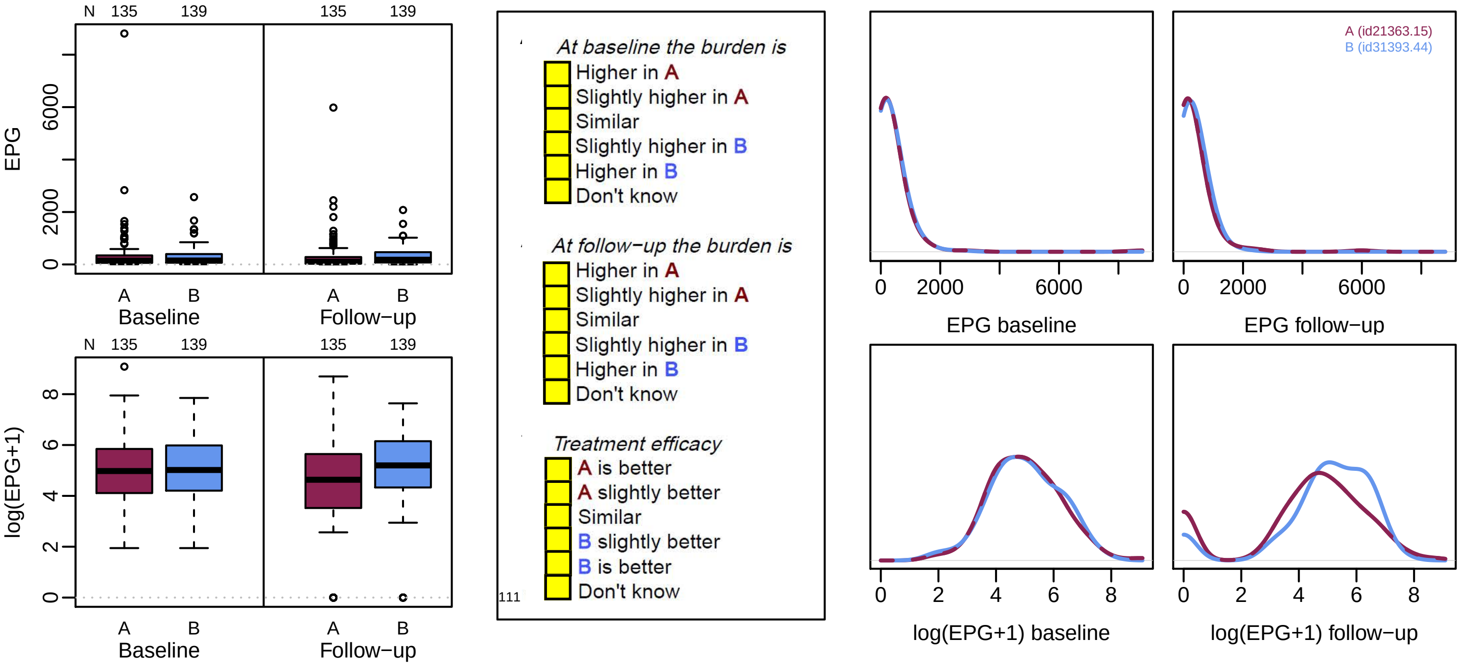
N 135 139 135 139
EPG
Baseline
Follow−up
log(EPG+1)
At baseline the burden is
Higher in A
Slightly higher in A
Similar
Slightly higher in B
Higher in B
Don't know
At follow−up the burden is
Higher in A
Slightly higher in A
Similar
Slightly higher in B
Higher in B
Don't know
Treatment efficacy
A is better
A slightly better
Similar
B slightly better
B is better
Don't know
111
A (id21363.15)
B (id31393.44)
EPG baseline
EPG follow−up
log(EPG+1) baseline
log(EPG+1) follow−up

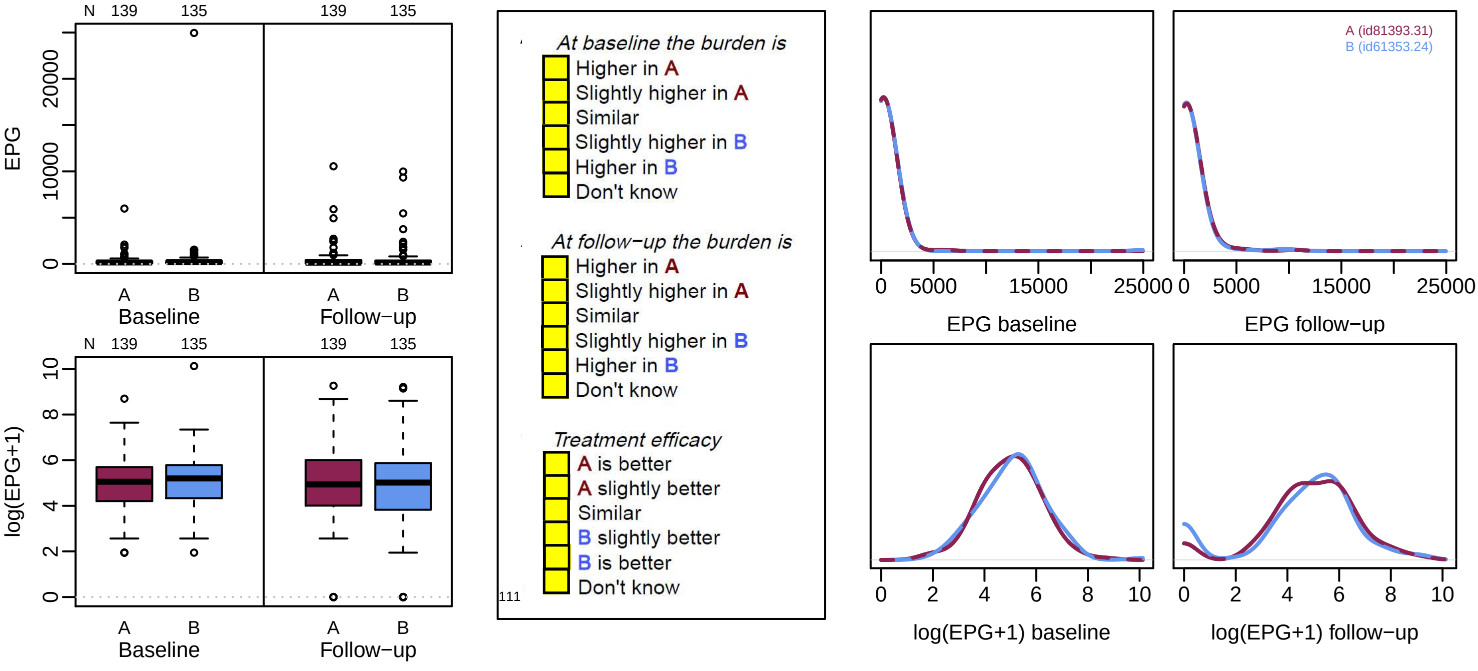
N 139 135 139 135
EPG
Baseline
Follow−up
log(EPG+1)
At baseline the burden is
Higher in A
Slightly higher in A
Similar
Slightly higher in B
Higher in B
Don't know
At follow−up the burden is
Higher in A
Slightly higher in A
Similar
Slightly higher in B
Higher in B
Don't know
Treatment efficacy
A is better
A slightly better
Similar
B slightly better
B is better
Don't know
111
A (id81393.31)
B (id61353.24)
EPG baseline
EPG follow−up
log(EPG+1) baseline
log(EPG+1) follow−up

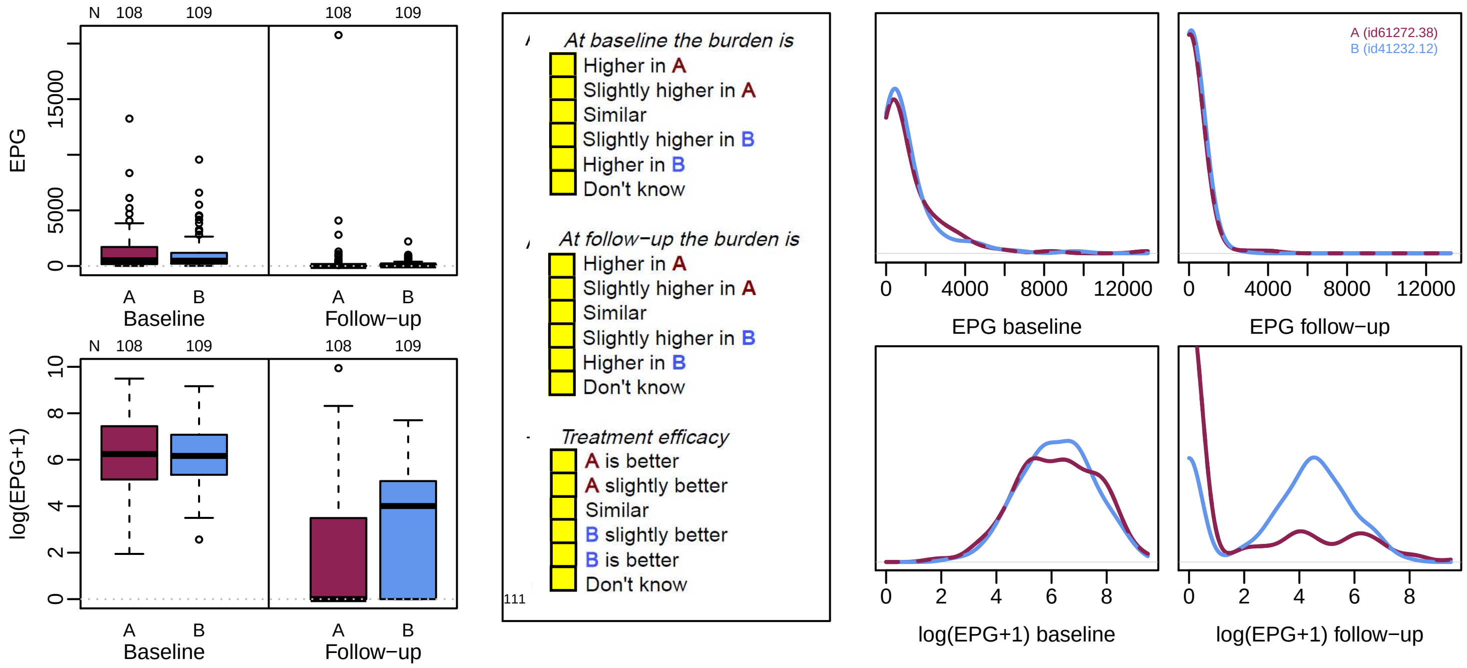
N 108 109 108 109
EPG
Baseline
Follow−up
log(EPG+1)
At baseline the burden is
Higher in A
Slightly higher in A
Similar
Slightly higher in B
Higher in B
Don't know
At follow−up the burden is
Higher in A
Slightly higher in A
Similar
Slightly higher in B
Higher in B
Don't know
Treatment efficacy
A is better
A slightly better
Similar
B slightly better
B is better
Don't know
111
A (id61272.38)
B (id41232.12)
EPG baseline
EPG follow−up
log(EPG+1) baseline
log(EPG+1) follow−up

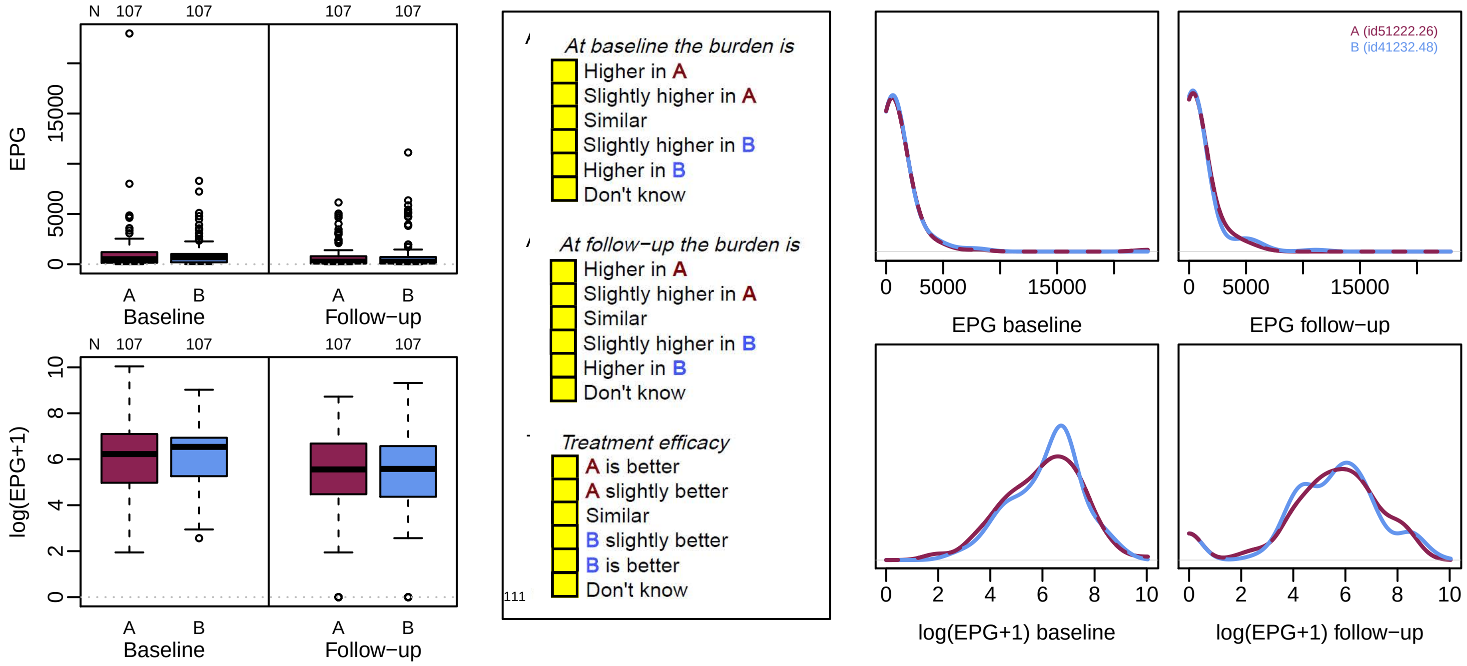
N 107 107 107 107
EPG
Baseline
Follow−up
log(EPG+1)
At baseline the burden is
Higher in A
Slightly higher in A
Similar
Slightly higher in B
Higher in B
Don't know
At follow−up the burden is
Higher in A
Slightly higher in A
Similar
Slightly higher in B
Higher in B
Don't know
Treatment efficacy
A is better
A slightly better
Similar
B slightly better
B is better
Don't know
111
A (id51222.26)
B (id41232.48)
EPG baseline
EPG follow−up
log(EPG+1) baseline
log(EPG+1) follow−up

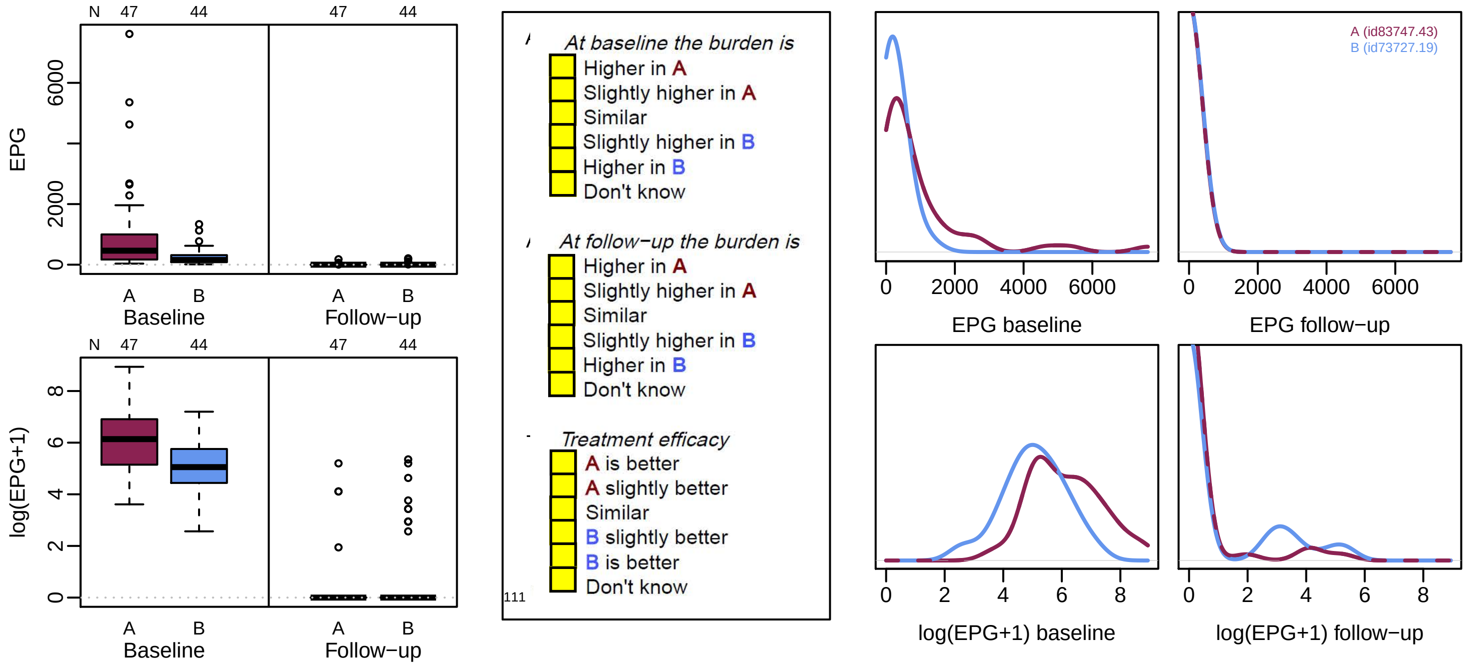
N 47 44 47 44
EPG
Baseline
Follow−up
log(EPG+1)
At baseline the burden is
Higher in A
Slightly higher in A
Similar
Slightly higher in B
Higher in B
Don't know
At follow−up the burden is
Higher in A
Slightly higher in A
Similar
Slightly higher in B
Higher in B
Don't know
Treatment efficacy
A is better
A slightly better
Similar
B slightly better
B is better
Don't know
111
A (id83747.43)
B (id73727.19)
EPG baseline
EPG follow−up
log(EPG+1) baseline
log(EPG+1) follow−up

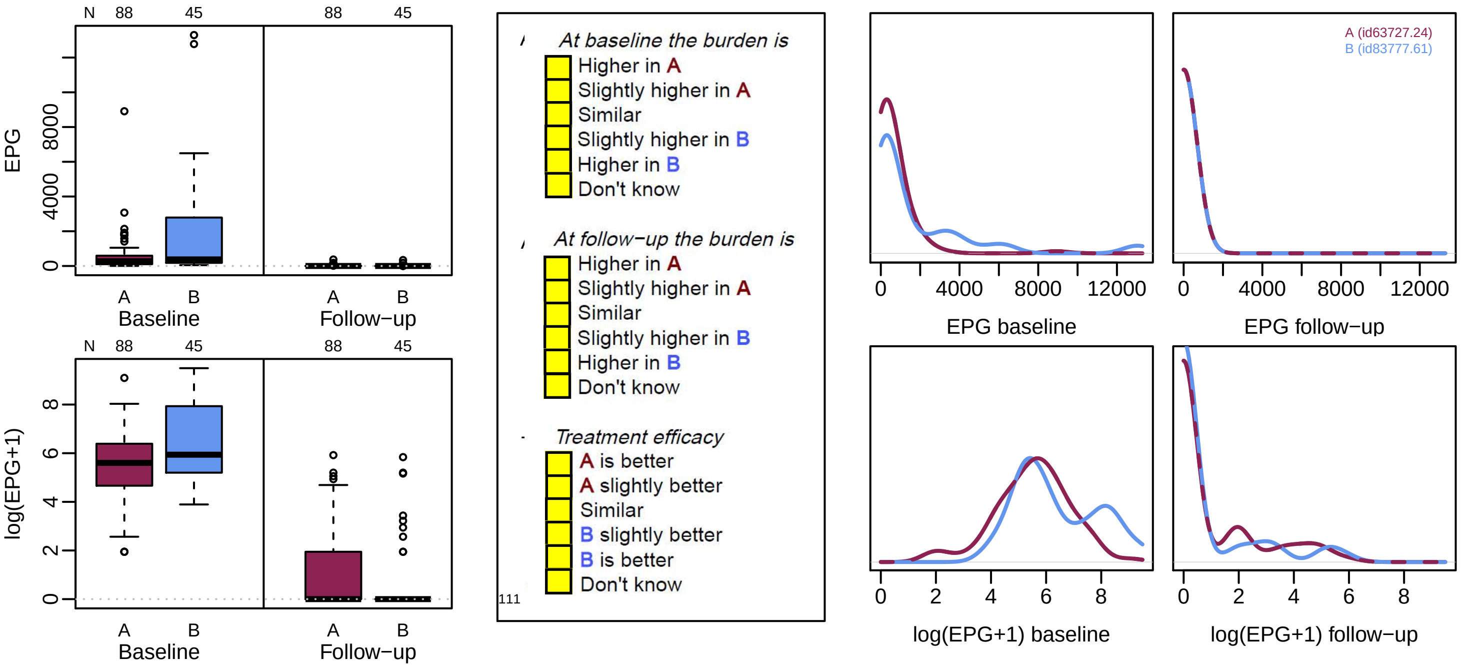
N 88 45 88 45
EPG
0 4000 8000
A B Baseline
A B Follow−up
log(EPG+1)
0 2 4 6 8
At baseline the burden is
Higher in A
Slightly higher in A
Similar
Slightly higher in B
Higher in B
Don't know
At follow−up the burden is
Higher in A
Slightly higher in A
Similar
Slightly higher in B
Higher in B
Don't know
Treatment efficacy
A is better
A slightly better
Similar
B slightly better
B is better
Don't know
111
A (id63727.24)
B (id83777.61)
0 4000 8000 12000
EPG baseline
EPG follow−up
log(EPG+1) baseline
log(EPG+1) follow−up

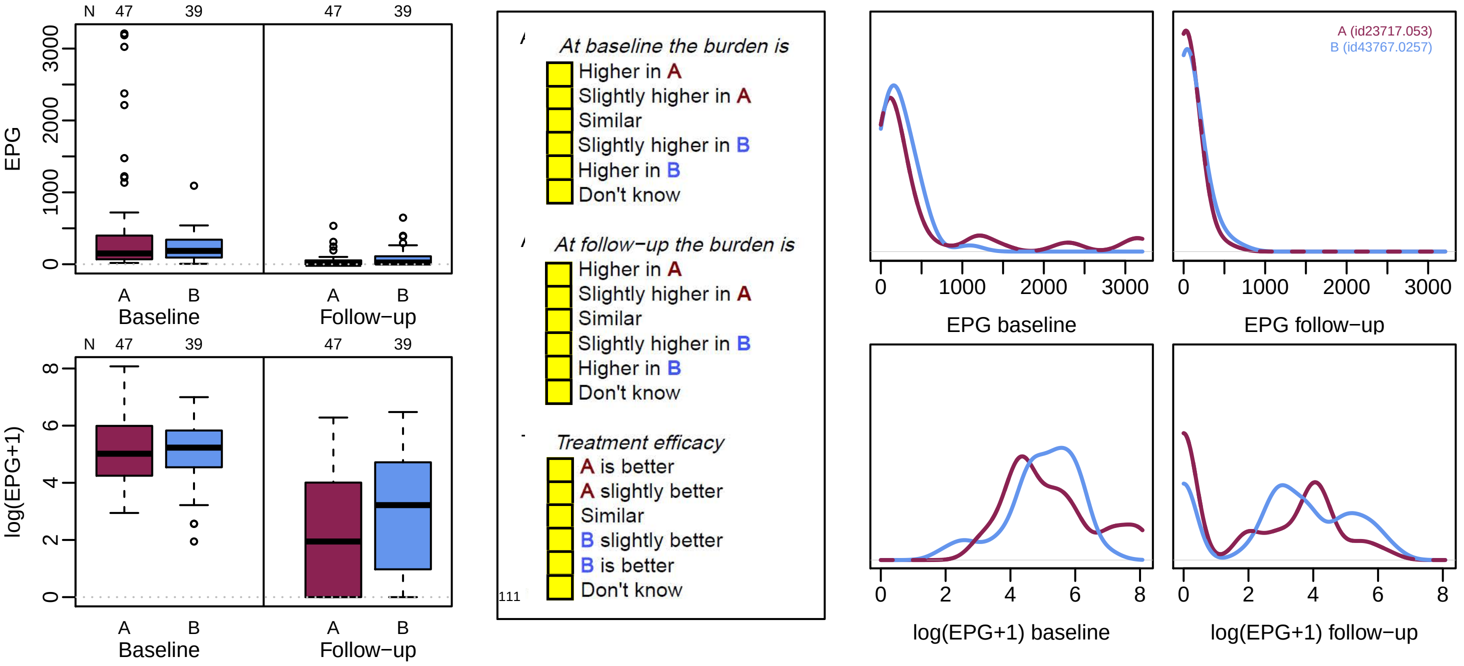
N 47 39 47 39
EPG
0 1000 2000 3000
A B Baseline
A B Follow−up
log(EPG+1)
0 2 4 6 8
At baseline the burden is
Higher in A
Slightly higher in A
Similar
Slightly higher in B
Higher in B
Don't know
At follow−up the burden is
Higher in A
Slightly higher in A
Similar
Slightly higher in B
Higher in B
Don't know
Treatment efficacy
A is better
A slightly better
Similar
B slightly better
B is better
Don't know
111
A (id23717.053)
B (id43767.0257)
EPG baseline
EPG follow−up
log(EPG+1) baseline
log(EPG+1) follow−up

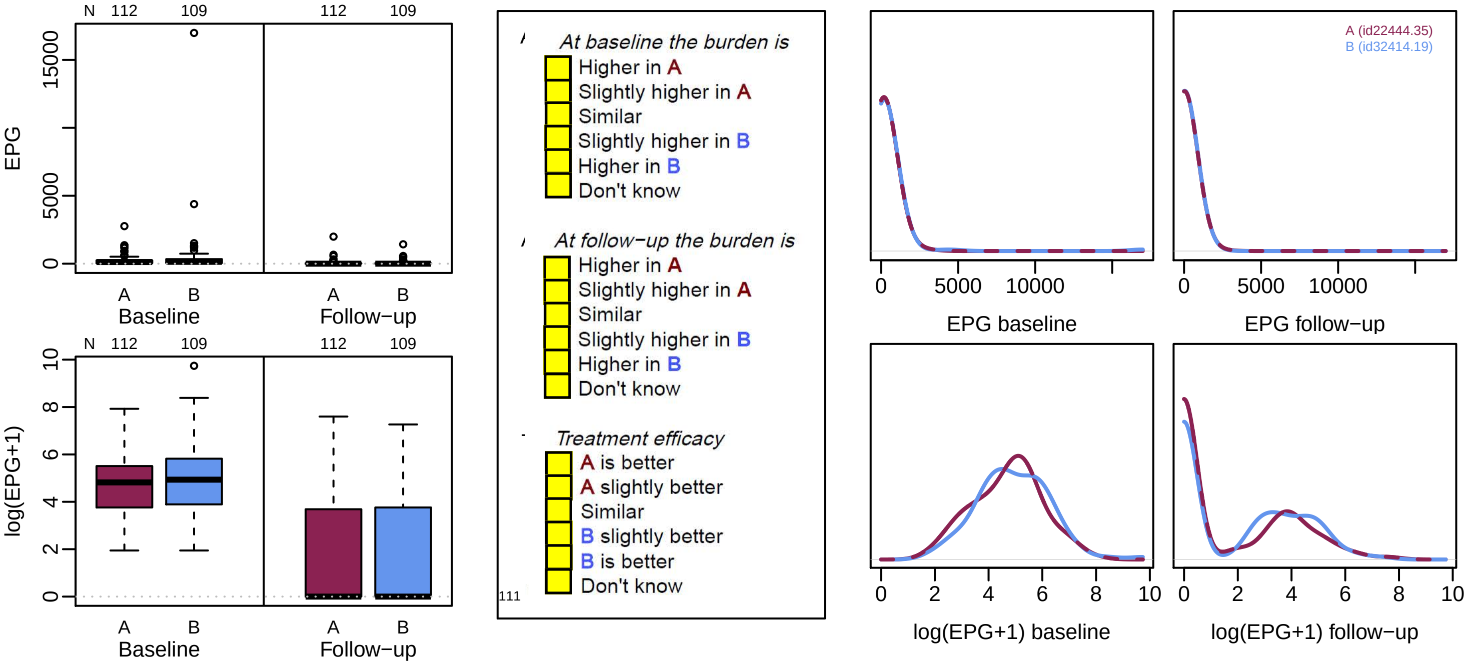
N 112 109 112 109
EPG
0 5000 15000
A B Baseline
A B Follow−up
log(EPG+1)
0 2 4 6 8 10
At baseline the burden is
Higher in A
Slightly higher in A
Similar
Slightly higher in B
Higher in B
Don't know
At follow−up the burden is
Higher in A
Slightly higher in A
Similar
Slightly higher in B
Higher in B
Don't know
Treatment efficacy
A is better
A slightly better
Similar
B slightly better
B is better
Don't know
111
A (id22444.35)
B (id32414.19)
0 5000 10000
EPG baseline
EPG follow−up
log(EPG+1) baseline
log(EPG+1) follow−up

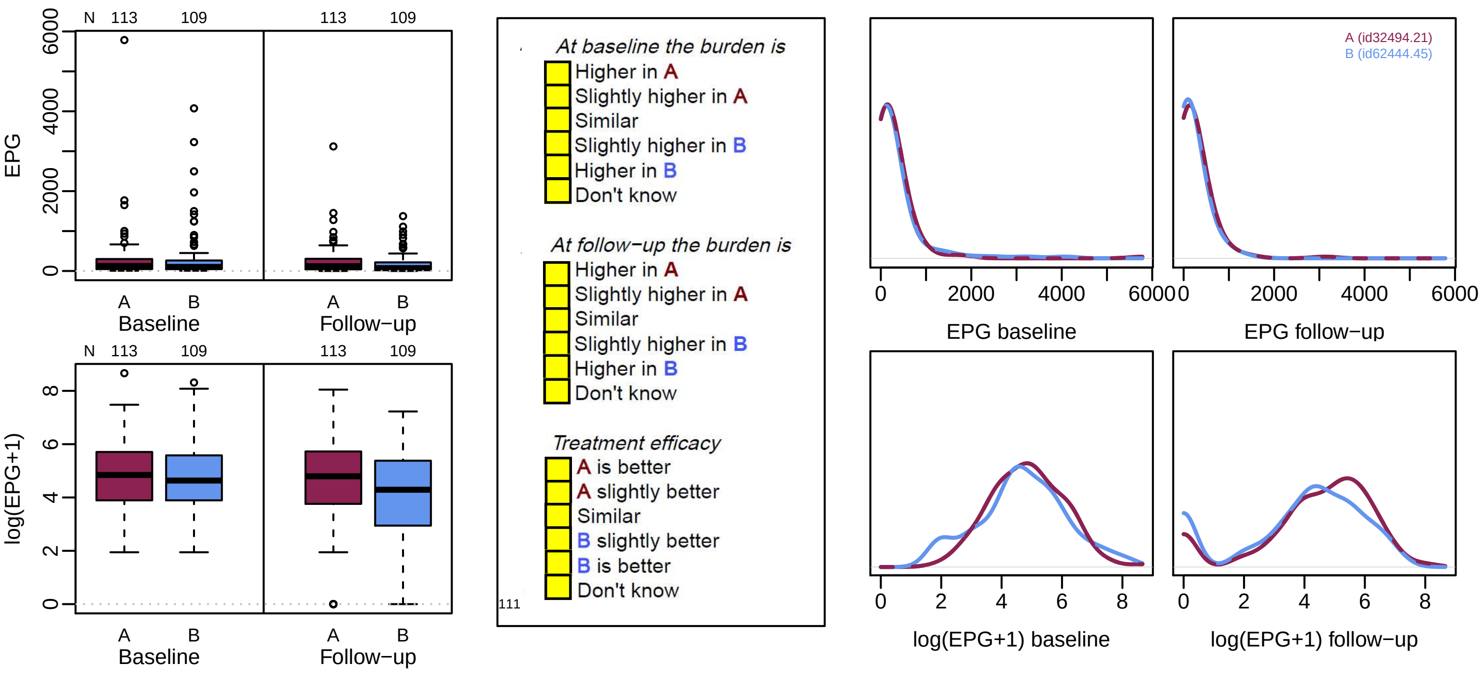

N 113 109 113 109
EPG
log(EPG+1)
A B A B
Baseline
Follow−up
At baseline the burden is
Higher in A
Slightly higher in A
Similar
Slightly higher in B
Higher in B
Don't know
At follow−up the burden is
Higher in A
Slightly higher in A
Similar
Slightly higher in B
Higher in B
Don't know
Treatment efficacy
A is better
A slightly better
Similar
B slightly better
B is better
Don't know
111
A (id32494.21)
B (id62444.45)
EPG baseline
EPG follow−up
log(EPG+1) baseline
log(EPG+1) follow−up

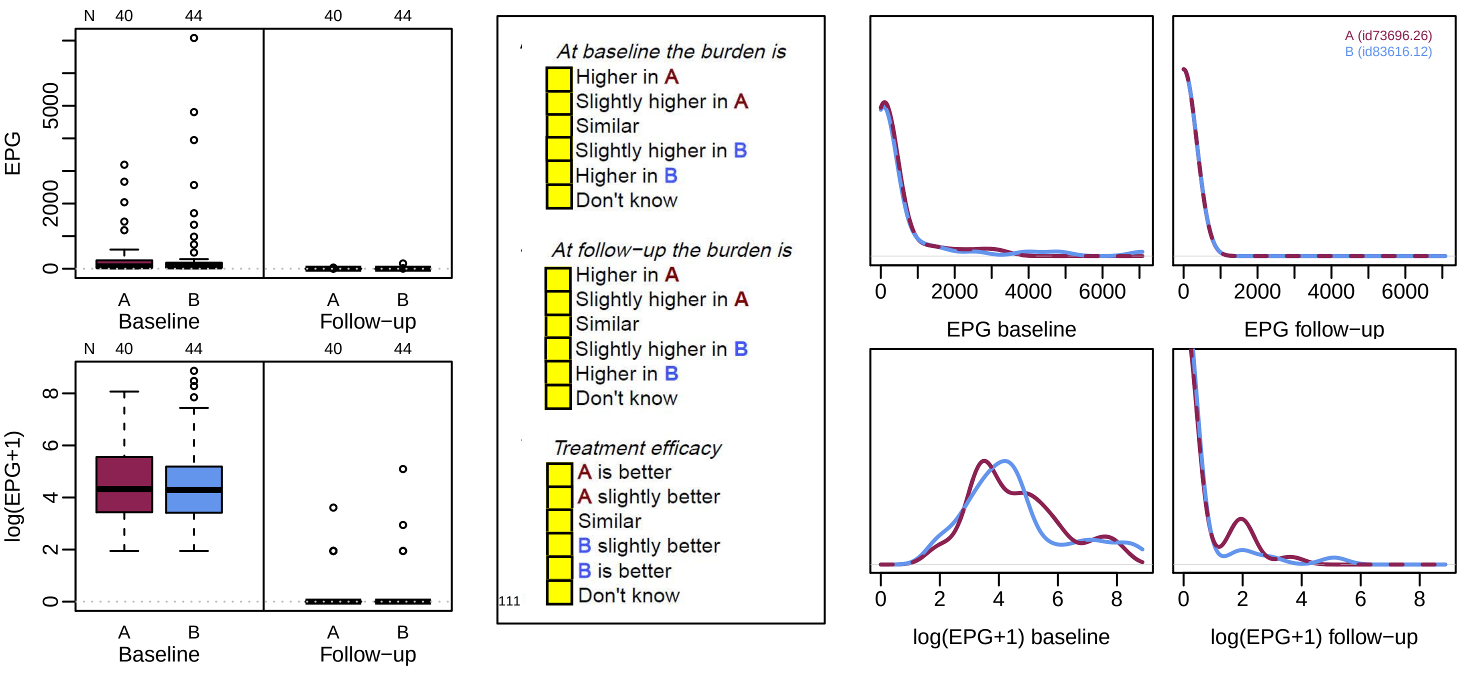

N 40 44 40 44
EPG
log(EPG+1)
A B A B
Baseline
Follow−up
At baseline the burden is
Higher in A
Slightly higher in A
Similar
Slightly higher in B
Higher in B
Don't know
At follow−up the burden is
Higher in A
Slightly higher in A
Similar
Slightly higher in B
Higher in B
Don't know
Treatment efficacy
A is better
A slightly better
Similar
B slightly better
B is better
Don't know
111
A (id73696.26)
B (id83616.12)
EPG baseline
EPG follow−up
log(EPG+1) baseline
log(EPG+1) follow−up

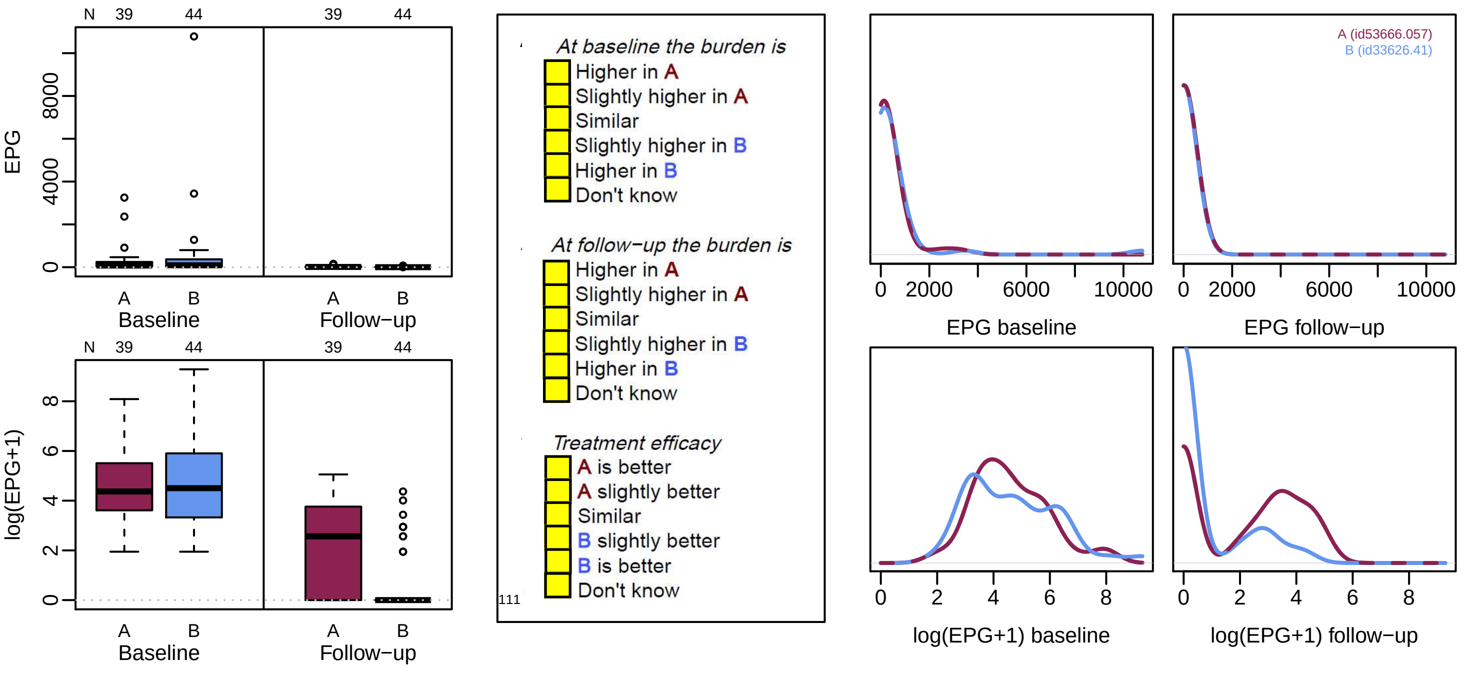

N 39 44 39 44
EPG
log(EPG+1)
A B A B
Baseline
Follow−up
At baseline the burden is
Higher in A
Slightly higher in A
Similar
Slightly higher in B
Higher in B
Don't know
At follow−up the burden is
Higher in A
Slightly higher in A
Similar
Slightly higher in B
Higher in B
Don't know
Treatment efficacy
A is better
A slightly better
Similar
B slightly better
B is better
Don't know
111
A (id53666.057)
B (id33626.41)
EPG baseline
EPG follow−up
log(EPG+1) baseline
log(EPG+1) follow−up

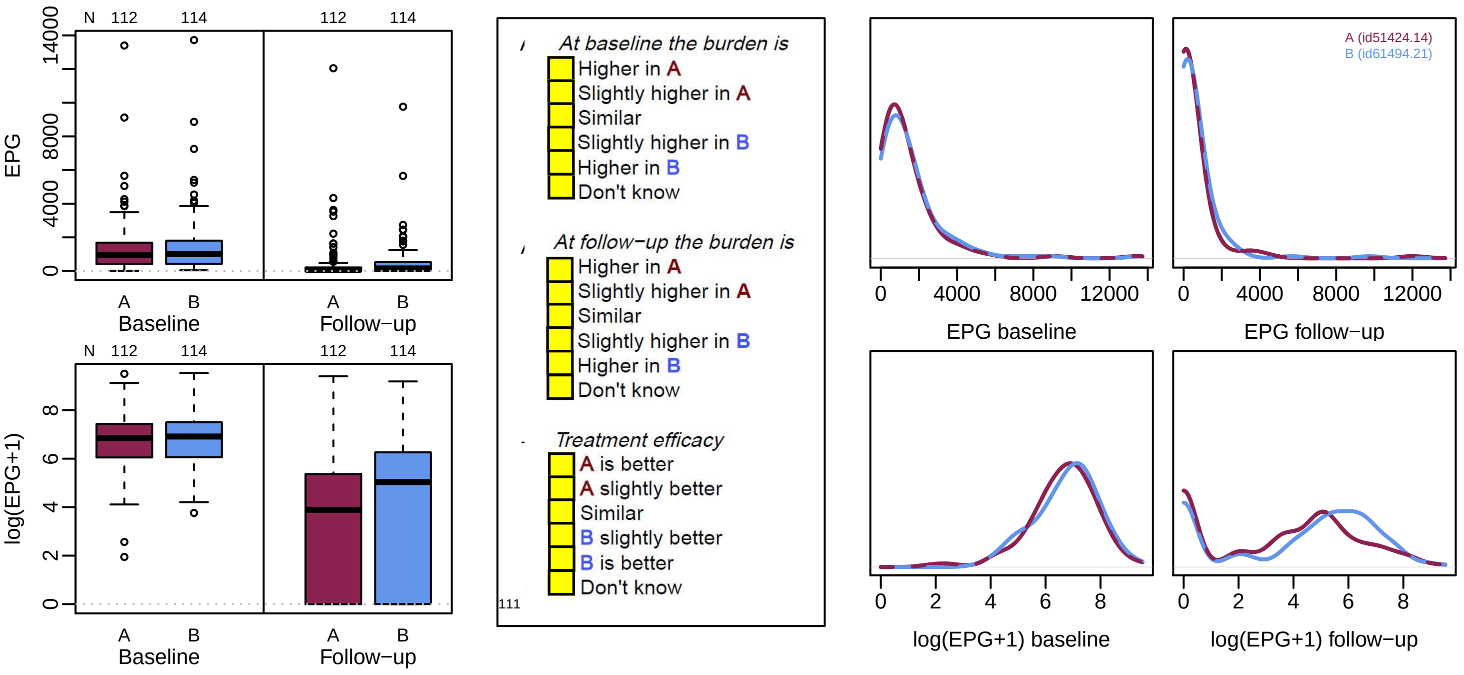
N 112 114 112 114
EPG
0 4000 8000 14000
A B A B
Baseline Follow−up
N 112 114 112 114
log(EPG+1)
0 2 4 6 8
A B A B
Baseline Follow−up
At baseline the burden is
Higher in A
Slightly higher in A
Similar
Slightly higher in B
Higher in B
Don't know
At follow−up the burden is
Higher in A
Slightly higher in A
Similar
Slightly higher in B
Higher in B
Don't know
Treatment efficacy
A is better
A slightly better
Similar
B slightly better
B is better
Don't know
111
A (id51424.14)
B (id61494.21)
0 4000 8000 12000
EPG baseline
0 4000 8000 12000
EPG follow−up
0 2 4 6 8
log(EPG+1) baseline
0 2 4 6 8
log(EPG+1) follow−up

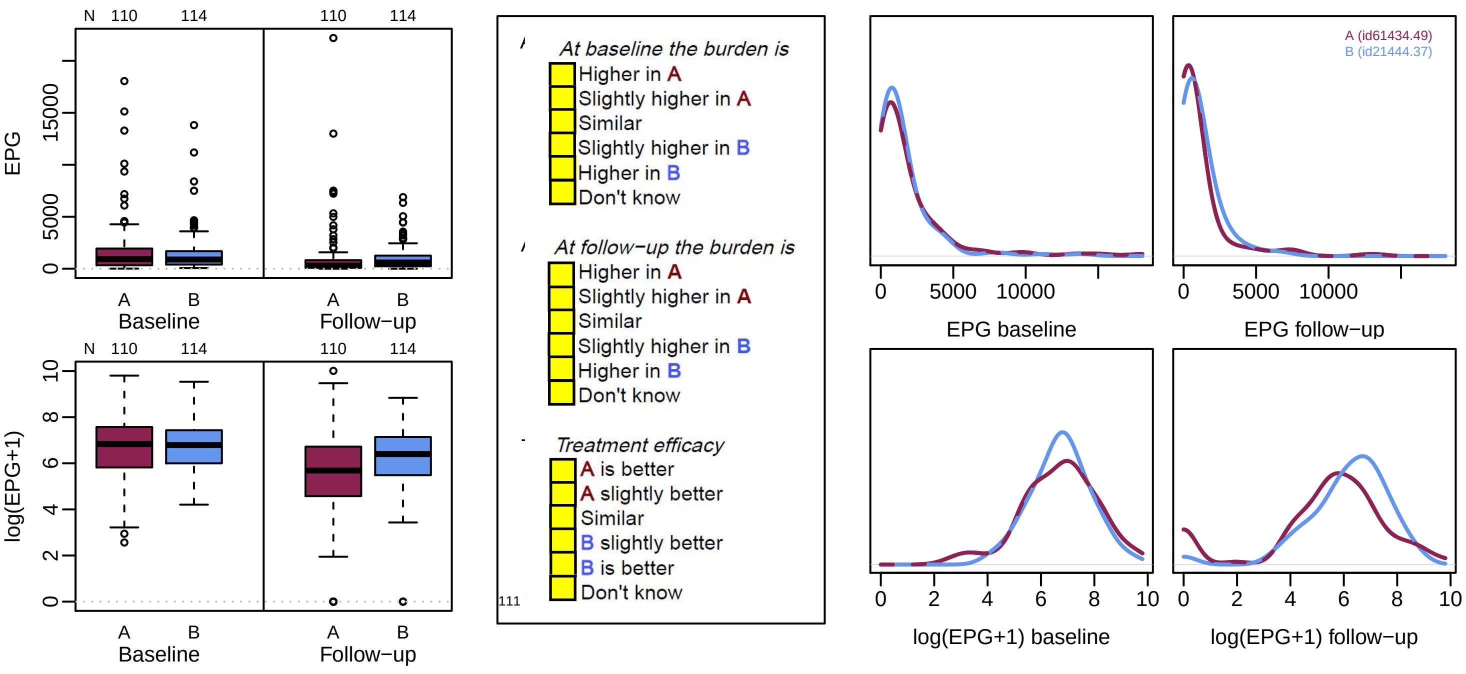
N 110 114 110 114
EPG
0 5000 15000
A B A B
Baseline Follow−up
N 110 114 110 114
log(EPG+1)
0 2 4 6 8 10
A B A B
Baseline Follow−up
At baseline the burden is
Higher in A
Slightly higher in A
Similar
Slightly higher in B
Higher in B
Don't know
At follow−up the burden is
Higher in A
Slightly higher in A
Similar
Slightly higher in B
Higher in B
Don't know
Treatment efficacy
A is better
A slightly better
Similar
B slightly better
B is better
Don't know
111
A (id61434.49)
B (id21444.37)
0 5000 10000
EPG baseline
0 5000 10000
EPG follow−up
0 2 4 6 8 10
log(EPG+1) baseline
0 2 4 6 8 10
log(EPG+1) follow−up

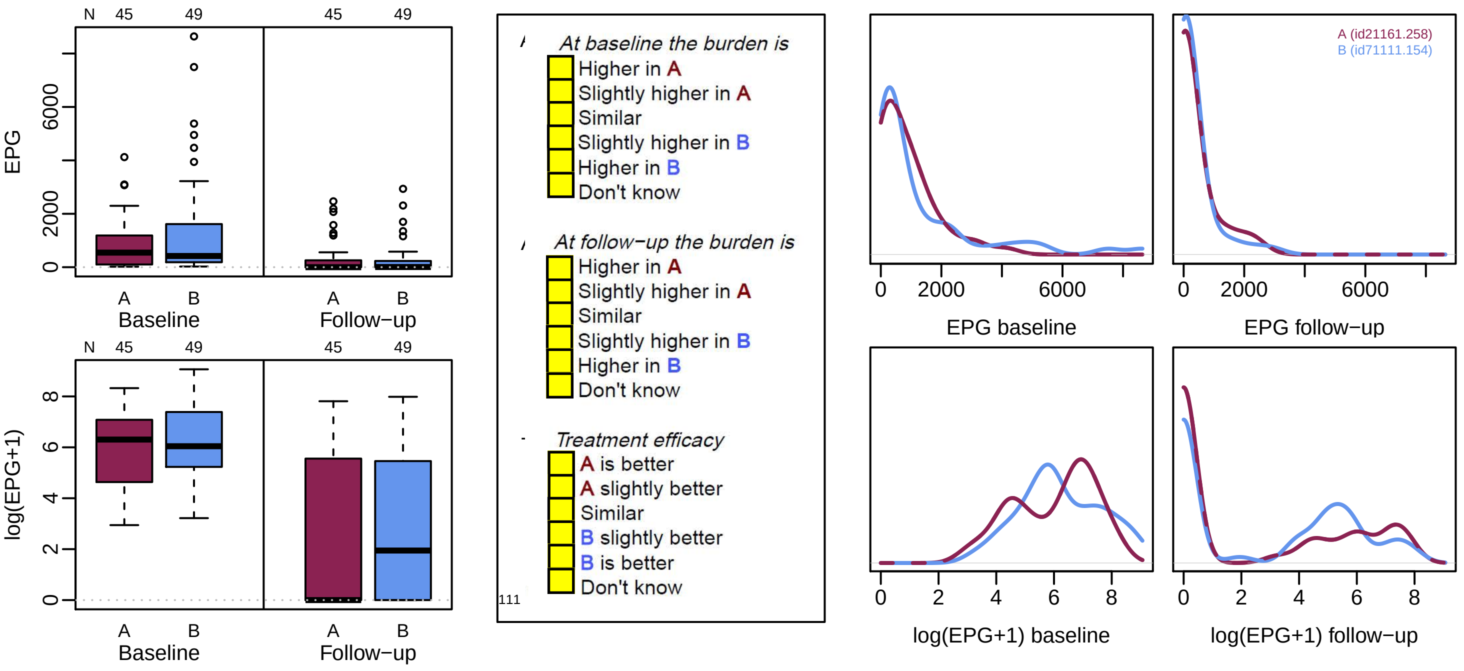
N 45 49 45 49
EPG
0 2000 6000
A B A B
Baseline Follow−up
N 45 49 45 49
log(EPG+1)
0 2 4 6 8
A B A B
Baseline Follow−up
At baseline the burden is
Higher in A
Slightly higher in A
Similar
Slightly higher in B
Higher in B
Don't know
At follow−up the burden is
Higher in A
Slightly higher in A
Similar
Slightly higher in B
Higher in B
Don't know
Treatment efficacy
A is better
A slightly better
Similar
B slightly better
B is better
Don't know
111
A (id21161.258)
B (id71111.154)
0 2000 6000
EPG baseline
0 2000 6000
EPG follow−up
0 2 4 6 8
log(EPG+1) baseline
0 2 4 6 8
log(EPG+1) follow−up

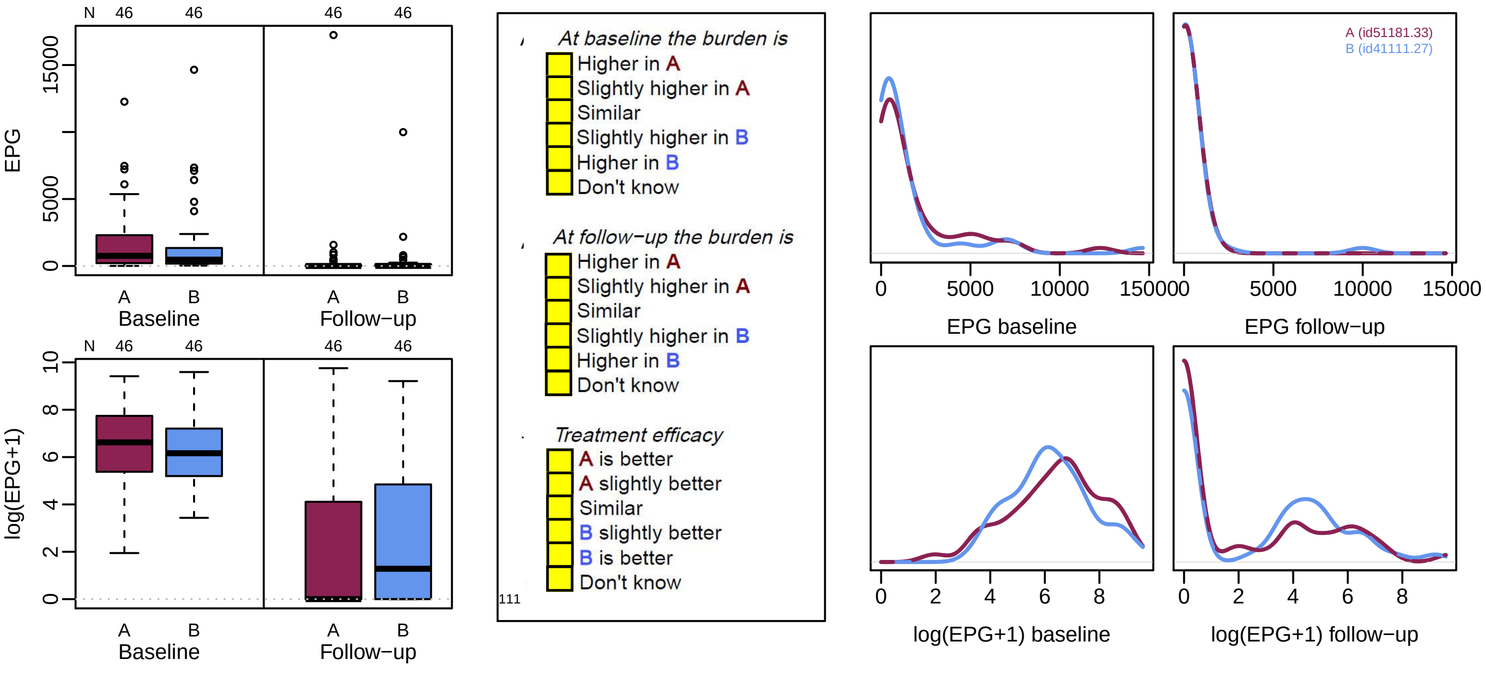
N 46 46 46 46
EPG
Baseline
Follow−up
log(EPG+1)
At baseline the burden is
Higher in A
Slightly higher in A
Similar
Slightly higher in B
Higher in B
Don't know
At follow−up the burden is
Higher in A
Slightly higher in A
Similar
Slightly higher in B
Higher in B
Don't know
Treatment efficacy
A is better
A slightly better
Similar
B slightly better
B is better
Don't know
111
A (id51181.33)
B (id41111.27)
EPG baseline
EPG follow−up
log(EPG+1) baseline
log(EPG+1) follow−up

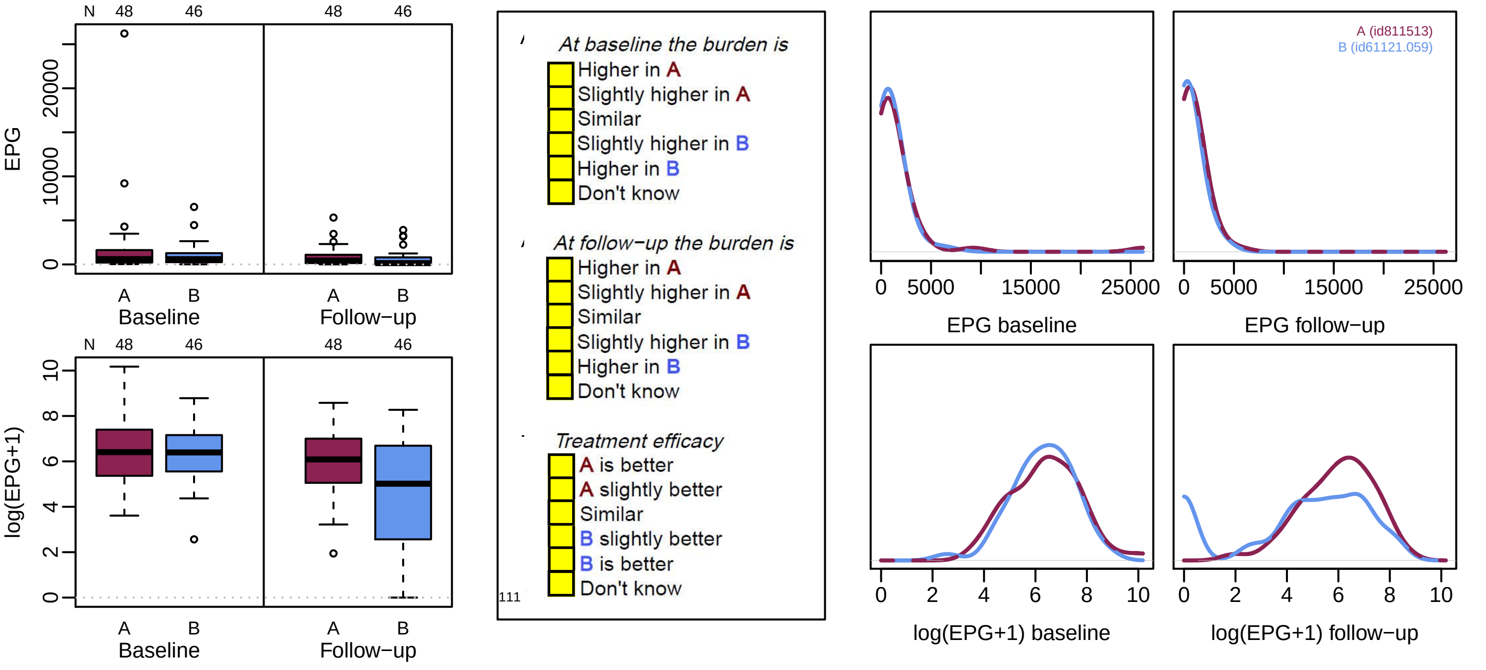
N 48 46 48 46
EPG
Baseline
Follow−up
log(EPG+1)
At baseline the burden is
Higher in A
Slightly higher in A
Similar
Slightly higher in B
Higher in B
Don't know
At follow−up the burden is
Higher in A
Slightly higher in A
Similar
Slightly higher in B
Higher in B
Don't know
Treatment efficacy
A is better
A slightly better
Similar
B slightly better
B is better
Don't know
111
A (id811513)
B (id61121.059)
EPG baseline
EPG follow−up
log(EPG+1) baseline
log(EPG+1) follow−up
