# Supplementary material for: One mean to rule them all? The arithmetic mean based egg reduction rate can be misleading when estimating anthelminthic drug efficacy in clinical trials
Source: PLoS Negl Trop Dis. 2020 Apr 8;14(4):e0008185. doi: 10.1371/journal.pntd.0008185 (PMC7170292; doi:10.1371/journal.pntd.0008185)

Guidance regarding figure relationship and interpretation
Example: Follow-up

Fig 3

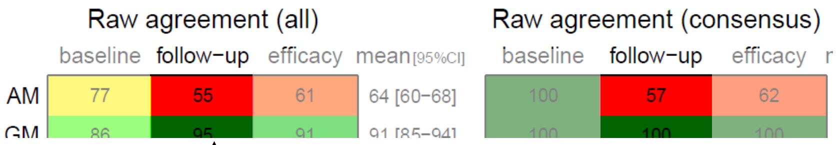

Agreement	>50% votes: B higher; absolute majority	A more often but <50% (excl. ■) simple majority	>50% votes: A higher; absolute majority
**All studies**	B higher	A higher	A higher
**Consensus**	B higher	Excluded	A higher

Arithmetic mean

**Experts**	N studies A>B	N studies A<B
N studies A>B	4	4
N studies A<B	6	8

Raw ageement: (4+8)/22 = 55%

Fig 2

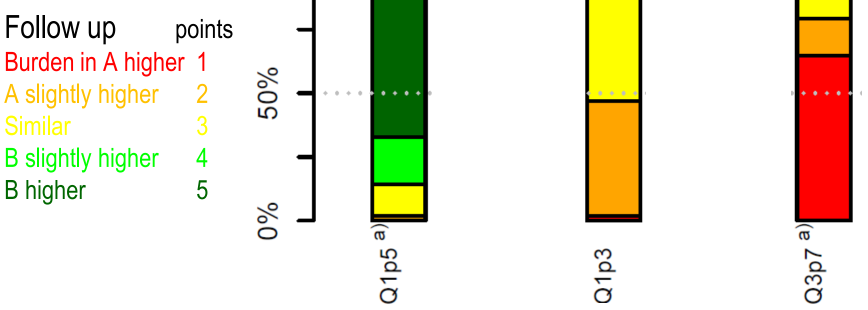

**Scores**	1×2,6×3,9×4,33×5 mean = 4.5	1×2,22×2,25×3,1×4 mean = 2.5	31×1,7×2,10×3 mean = 1.6

mean > 3: reverse
4.5 = 1.5
■ = ■

Fig 4

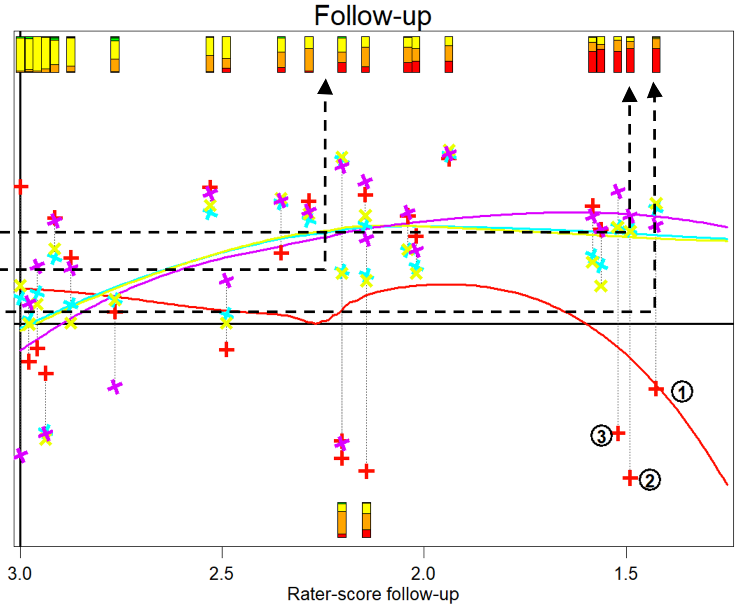

Supplement: S2 File — (PDF) [file pntd.0008185.s002.pdf]
