# Supplementary material for: One mean to rule them all? The arithmetic mean based egg reduction rate can be misleading when estimating anthelminthic drug efficacy in clinical trials
Source: PLoS Negl Trop Dis. 2020 Apr 8;14(4):e0008185. doi: 10.1371/journal.pntd.0008185 (PMC7170292; doi:10.1371/journal.pntd.0008185)

Supplementary appendix S2 File

Table of contents

Table S1. Cure rates and egg counts for the 7 included studies 2

Figure S1. Sensitivity analysis - Judgment of 49 raters with respect to the egg burden and treatment efficacy of 2 clinical trial arms 3

Figure S2. Sensitivity analysis - percentage agreement between experts and different means 4

Figure S3. Sensitivity analysis - relationship between the calculated difference among 2 trial arms estimated by different means and experts' rating scores 5

Figure S4. Sensitivity analysis - relationship of raters and means and cure rates 6

Figure S5. Sensitivity analysis - Relationship between difference in ERR and cure rates using weights and alternative scaling 7

Figure S6. Agreement among different measures of central tendency 8

Table S1. Cure rates and egg counts for the 7 included studies

The Sayasone et al. 2016 publication consists of 2 distinct trials (adults and children).

Study	Treatment arm	N	N pos FU	Cure rate %	EG counts BL Med	EG counts BL IQR	EG counts FU Med	EG counts FU IQR
**Hookworm**								
Moser et al. 2016	Placebo	13	11	15.4	138	48-246	126	48-240
	OxP, 5 mg/kg	17	9	47.1	48	24-96	6	0-108
	OxP, 10 mg/kg[a)]	13	3	23.1	48	24-162	60	18-186
	OxP, 15 mg/kg	17	11	35.3	36	30-84	30	0-126
	OxP, 20 mg/kg	18	12	33.3	48	36-180	51	0-90
	OxP, 25 mg/kg	18	13	27.8	72	36-168	51	0-240
	OxP, 30 mg/kg	19	16	15.8	102	30-270	96	12-366
Speich et al. 2014	Alb-OxP	109	53	51.4	138	48-336	0	0-42
	OxP, 20mg/kg	113	101	2.9	126	48-300	120	42-306
	Alb	112	45	59.8	123	42-246	0	0-39
	Meb	109	90	17.4	102	48-264	72	18-216
Knopp et al. 2010	Alb-Iver	30	10	66.7	69	42-144	0	0-24
	Alb	39	16	59.0	60	18-156	0	0-30
	Meb-Iver	35	25	25.7	36	12-240	48	0-120
	Meb	34	22	35.3	48	18-162	18	0-96
Opisthochis viverrini								
Sayasone et al. 2016	Trb 50mg	39	23	41.0	78	36-258	12	0-42
	Trb 100mg	44	4	90.9	72	30-186	0	0-0
	Trb 200mg	40	7	82.5	75	30-258	0	0-0
	Trb 400mg	44	10	77.3	90	27-369	0	0-0
	Trb 25mg	39	29	25.6	186	90-384	24	0-114
	Trb 50mg	47	27	42.6	150	66-402	6	0-54
	Trb 100mg	44	10	77.3	156	84-315	0	0-0
	Trb 200mg	88	24	72.7	270	105-594	0	0-0
	Trb 400mg	47	4	91.5	462	168-1020	0	0-0
	Trb 600mg	45	9	80.0	378	180-2790	0	0-0

Study	Treatment arm	N	N pos FU	Cure rate %	EG counts BL Med	EG counts BL IQR	EG counts FU Med	EG counts FU IQR
Trichuris trichiura								
Moser et al. 2016	Placebo	48	48	0.0	606	213-1632	438	156-1098
	OxP, 5 mg/kg	46	36	21.7	597	258-1284	150	12-804
	OxP, 10 mg/kg[a)]	49	38	22.4	660	216-1806	210	24-564
	OxP, 15 mg/kg	49	25	49.0	420	186-1614	6	0-234
	OxP, 20 mg/kg	46	23	50.0	474	180-1344	6	0-126
	OxP, 25 mg/kg	45	18	60.0	546	102-1188	0	0-258
	OxP, 30 mg/kg	46	19	58.7	753	21-2298	0	0-60
Speich et al. 2014	Alb-OxP	112	77	31.3	945	423-1686	48	0-213
	OxP, 20mg/kg	114	84	26.3	1005	426-1806	153	0-522
	Alb	114	111	2.6	888	402-1692	600	240-1260
	Meb	110	97	11.8	927	336-1944	294	96-828
Knopp et al. 2010	Alb-Iver	140	87	37.9	108	54-276	18	0-69
	Alb	132	119	9.8	156	69-300	108	42-252
	Meb-Iver	138	62	55.1	171	78-288	0	0-66
	Meb	138	112	18.8	132	66-294	75	24-210
Speich et al. 2015	Alb-Ivr	109	79	27.5	474	210-1182	54	0-160
	Alb-Meb	107	98	8.4	504	138-1212	258	84-822
	Alb-OxP	108	34	68.5	513	171-1710	0	0-33
	Meb	107	98	8.4	690	186-1074	264	72-720
Speich et al. 2012	Nita-Alb	135	113	16.3	144	60-354	102	30-282
	Alb	135	116	14.1	180	72-324	150	43-360
	Nita	139	130	6.5	156	66-300	138	54-408
	Placebo	139	127	8.6	150	66-408	180	72-474

[a)] This arm has been excluded because of an odd number of trial arms.

BL: baseline, FU: follow-up, Med: median, IQR: interquartile range

Figure S1. Sensitivity analysis - Judgment of 49 raters with respect to the egg burden and treatment efficacy of 2 clinical trial arms

To check the robustness of the results, we excluded comparisons with fewer than 30 observations per arm. The labels on the x-axis denote the page and question number (top, middle, bottom) in the example questionnaire presented in the appendix.

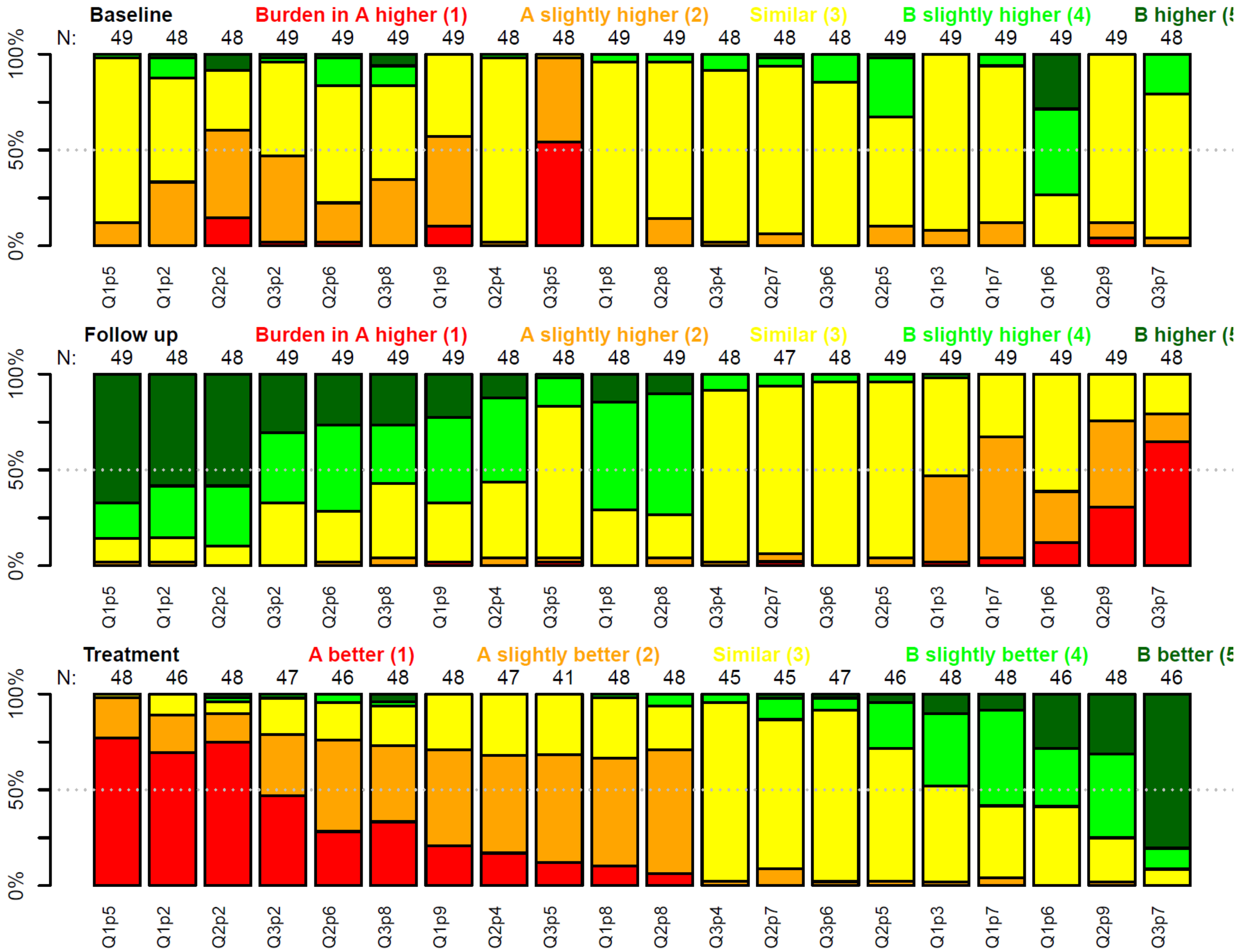

Figure S2. Sensitivity analysis - percentage agreement between experts and different means

To check the robustness of the results, we excluded comparisons with fewer than 30 observations per arm. Raw percentage agreement between experts' opinion and the calculated means for egg burden are at baseline and follow-up egg and drug efficacy (superiority of a certain trial arm). The rank denotes the rounded mean rank calculated separately for all comparisons and consensus comparisons. N (from left to right): 19, 19, 20, 4, 13, 15.

	Raw agreement (all)				Raw agreement (consensus)				
	baseline	follow-up	efficacy	mean	baseline	follow-up	efficacy	mean	rank
AM	74	53	65	64	100	62	67	76	25
GM	84	95	95	91	100	100	100	100	5
Hö0.1	89	95	95	93	100	100	100	100	4
Hö0.2	95	95	95	95	100	100	100	100	4
Hö0.3	89	95	90	91	100	100	93	98	8
Hö0.4	89	95	90	91	100	100	93	98	8
Hö0.5	84	89	95	90	100	92	93	95	10
Hö0.6	84	79	90	84	100	85	93	93	14
Hö0.7	79	74	85	79	100	77	93	90	18
Hö0.8	79	68	75	74	100	77	80	86	21
Hö0.9	79	58	70	69	100	62	73	78	23
Le0.1	68	95	85	83	75	100	87	87	18
Le0.2	68	95	90	84	75	100	93	89	15
Le0.3	68	95	90	84	75	100	93	89	15
Le0.4	79	95	90	88	75	100	93	89	13
Le0.5	84	95	95	91	100	100	93	98	7
Le0.6	84	89	95	90	100	100	93	98	8
Le0.7	89	84	90	88	100	92	93	95	11
Le0.8	84	79	85	83	100	85	93	93	15
Le0.9	79	68	75	74	100	77	80	86	21
Wi0.02	79	58	60	66	100	69	67	79	23
Wi0.04	79	79	90	83	75	92	93	87	17
Wi0.06	79	79	85	81	75	85	93	84	20
Wi0.08	79	84	90	84	75	85	93	84	18
Wi0.1	79	89	95	88	75	92	93	87	14
tr0.02	84	68	65	73	100	77	73	83	20
tr0.04	79	84	95	86	75	92	93	87	15
tr0.06	84	79	90	84	100	85	93	93	14
tr0.08	84	89	90	88	75	92	93	87	14
tr0.1	79	89	90	86	75	92	93	87	16

Figure S3. Sensitivity analysis - relationship between the calculated difference among 2 trial arms estimated by different means and experts' rating scores

To check the robustness of the results, we excluded comparisons with fewer than 30 observations per arm.

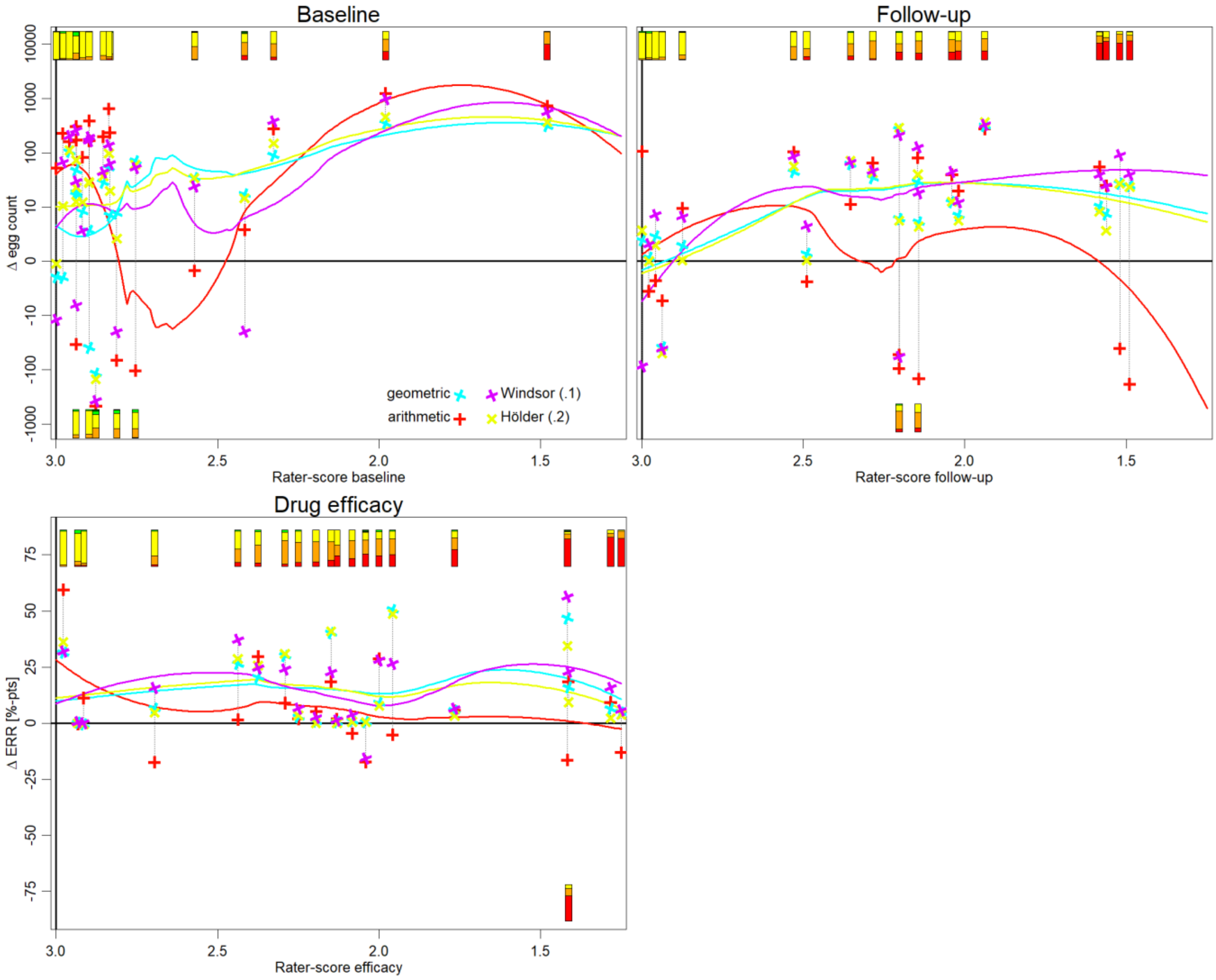

Scores that favoured arm B were converted [abs(score – 6)]. In this case the colours of the associated bar plots and the sign of the difference were reversed, too.

Figure S4. Sensitivity analysis - relationship of raters and means and cure rates

Differences between ERR and CRs in percentage points. Lines and shaded areas represent the loess smoothing line and the corresponding 95% confidence band. Grey crosses and the dotted line represent the experts' score and its corresponding loess smoothing line.

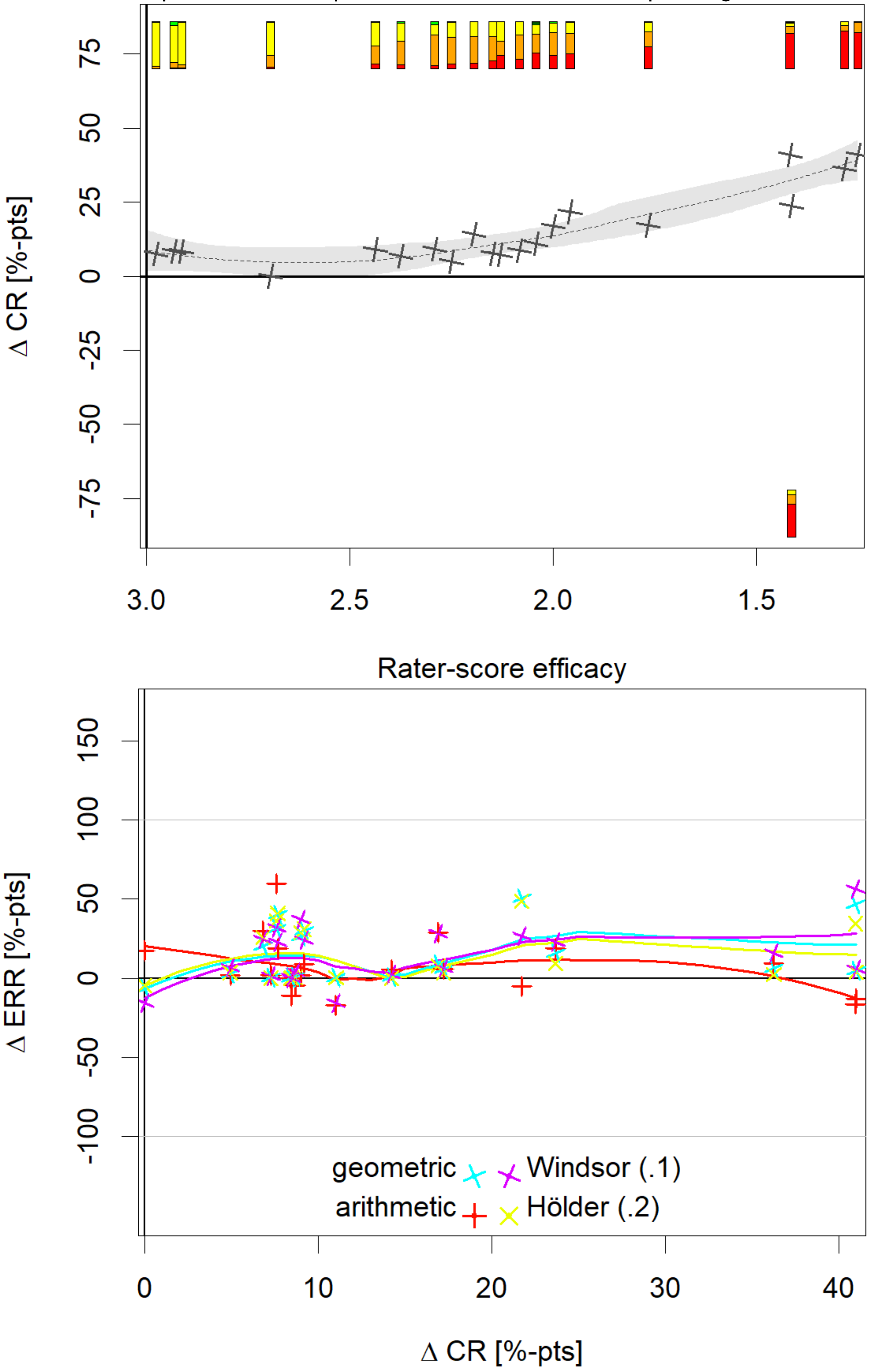

Figure S5. Sensitivity analysis - Relationship between difference in ERR and cure rates using weights and alternative scaling

Differences between ERR and cure rates in percentage points. Top panel: weighted lowess smoother (weights proportional to the number of subjects in the trial arms). Bottom panel: scaled differences in the ERRs (most extreme values were considered as minimum and maximum)

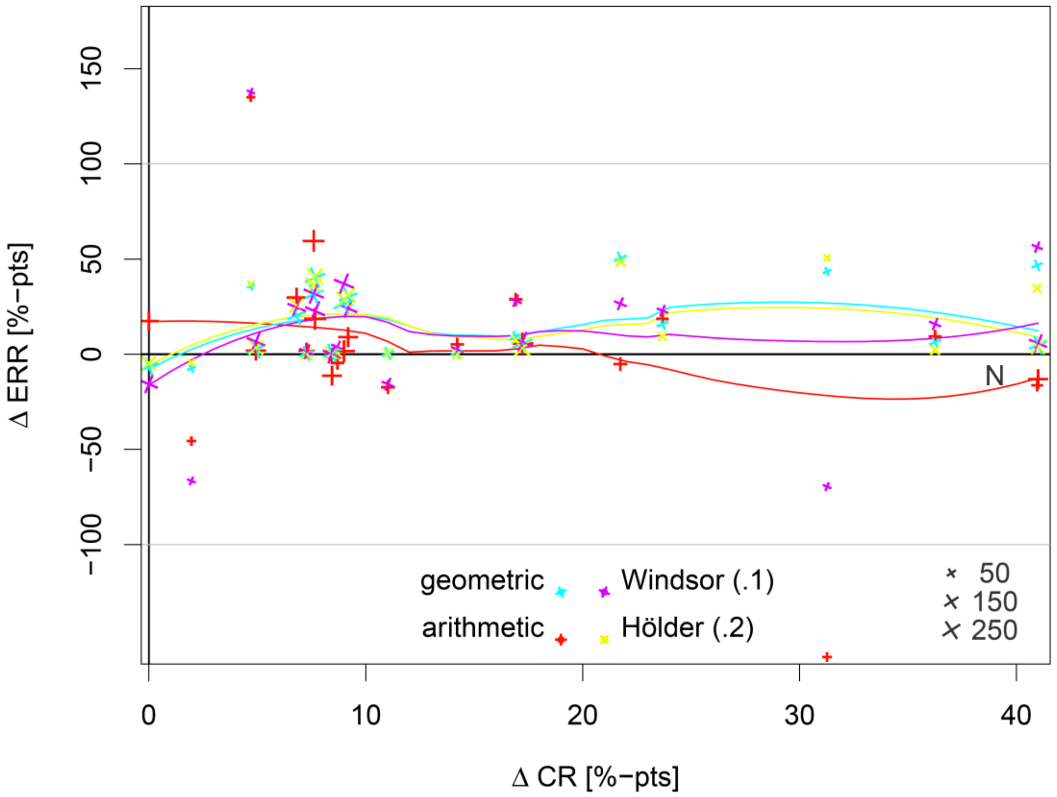

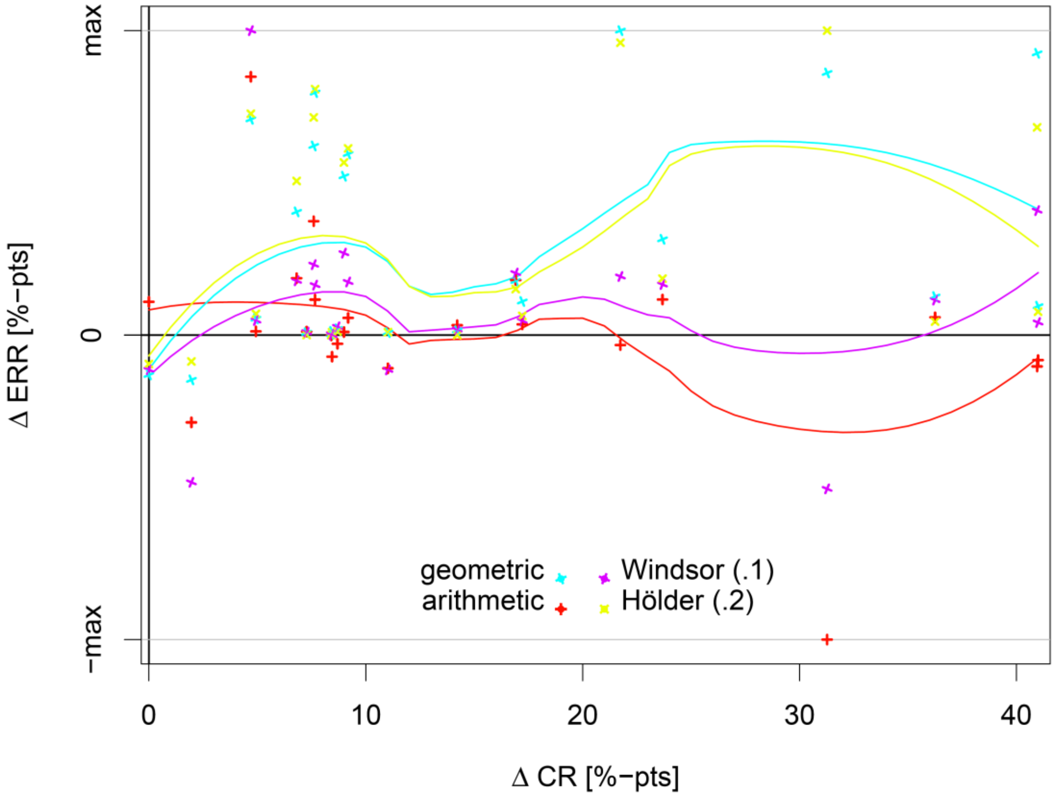

Figure S6. Agreement among different measures of central tendency

Raw agreement as the percentage of comparison where 2 different means are favoring the same trial arm. At baseline and follow-up egg burden where judged and finally drug efficacy (superiority of a certain trial arm). AM: arithmetic mean, GM: geometric mean, Hö: Hölder mean, Le: Lehmer mean, Wi: Winsorized mean, tr: truncated mean. Numbers behind abbreviations either indicate parameter p or the proportion discarded/replaced.

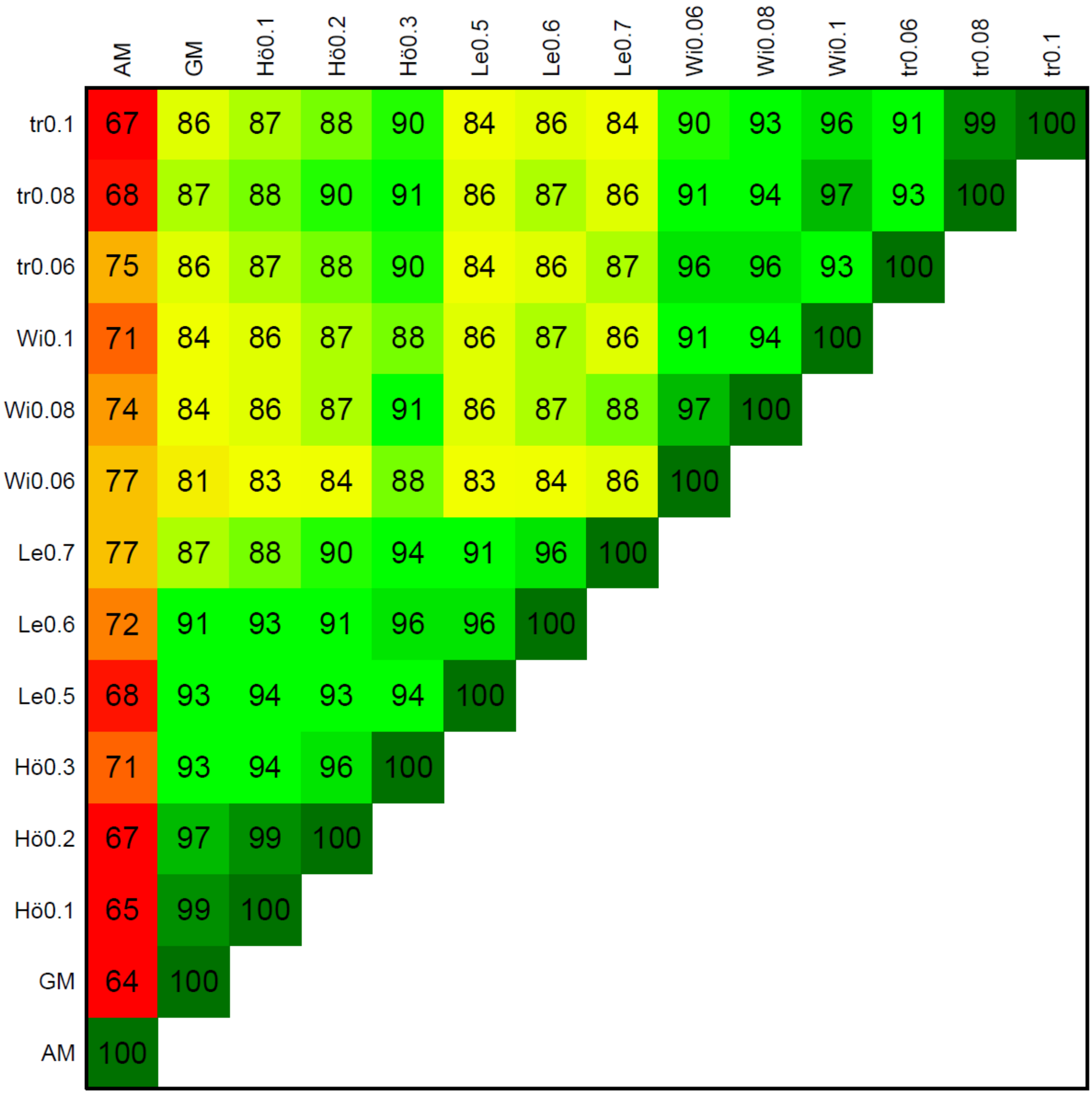

Supplement: S3 File — (PDF) [file pntd.0008185.s003.pdf]
