# Supplementary material for: One mean to rule them all? The arithmetic mean based egg reduction rate can be misleading when estimating anthelminthic drug efficacy in clinical trials
Source: PLoS Negl Trop Dis. 2020 Apr 8;14(4):e0008185. doi: 10.1371/journal.pntd.0008185 (PMC7170292; doi:10.1371/journal.pntd.0008185)
Supplement: Attachment — Submitted filename: PNTD-D-19-01393 LC.docx [file pntd.0008185.s004.docx]

Overall the manuscript is well-written, although inconsistencies in use of terminology and potential lack of detail (possibly as a result of the former) make it difficult to interpret the results in several instances. As a result, I was finding myself trawling back and forth between methods and results section to try and understand what was done and why and what it means. This doesn’t make for a very pleasant reading experience (2 hours from top to bottom of the paper) and is a distraction from the work’s content. Below, I provide some pointers on how to potentially improve this.

Supplement S1 (example questionnaire): it is very useful that the authors include this example. I’ve tried to assess a few of the trial arms, and realised how very challenging it actually is. Before having looked at the results, I was expecting a lot of variation between the experts’ answers for some of the comparisons. Was the first page all the information that the invited experts got? Or was there additional information? I am mentioning this because:

1) It is not clear to me what “graph 1-3” refers to in “The upper row (graph 1-3) shows eggs per gram.”. Should it say “graph 1-2”? I only see two panels (baseline and follow-up) in the top left box with the egg counts on the natural scale. It would be clearest if the panels are marked with a number of capital letter, and that these are used for reference.
2) “The box-plots present the min, Q1, median, Q3, max, dots represent outliers.” This seems inaccurate/incomplete; the ends of the whiskers on the box-plots cannot represent the minimum and maximum as there are also “outliers”. Most likely, if these box-plots where generated in R with *ggplot2* under the default settings, the whiskers represent *at most* the maximum, but no further than some constant times the IQR. And outliers are then defined as any data points outside this maximally allowed range of the whiskers. This needs to be clarified. Then again, maybe this is how it was presented to the experts…
3) What is the base of the logarithm being used for transformation of egg counts? Base 10 or is it the natural logarithm (or some other value)? The values on the y-axis of the logarithmic plots are uninterpretable this way. I am guessing the natural logarithm was used, based on looking at an outlier around 3000 epg that translated to a value of about 8 on the “log scale” (e^8 ~= 3000).

Although I am reasonably versed in applied stats and math, it is not clear to me what property is described by the equation (5) on line 160. Is *M(.)* the quantile function used, and does this mean that the result it returns will be the same if the input is entered in duplicate?

The argument to only apply truncation or winsorization to the right tail (high values) is not convincing. Egg counts of zero can be just as “extreme” in a sample where most egg counts are much higher. It is an inherent property of the negative binomial distribution of egg counts; both zeroes and high values can be considered extreme.

From the methods section (lines 184-185), it is not clear to me how the “raw percentage agreement” between the experts’ opinions and each of the mean metrics was calculated. What was considered agreement? In the preceding paragraph (lines 168-182), it is explained how the expert opinion was summarised over the whole group (in two ways), but I can’t tell whether or how these summaries where used for the calculation of the raw percentage agreement. Expert opinions were scored on a 5-point Likert scale; were the quantitative mean metrics and the differences between arms somehow translated to the 5-point Likert scale and then compared to the experts’ opinions? Or was the Likert scale dichotomised (if so, how?) and then compared to presence of a statistically significant difference in the quantitative mean metrics (ignoring the magnitude of a potential difference)? This needs a more explaining.

Although innovative and exciting (I would have definitely enjoyed to participate in the questionnaire), a weakness of the methodology used in this paper is that there is no way to benchmark how well the experts’ review of the trial result actually was, as the “true” differences between trials arms and differences in drug efficacy were not known. It may well be that our visual interpretation of data like these is inherently biased. The authors tried to address this by offering different visualisation methods, but it was up to each expert reviewer to choose what visualisation method to use primarily for their assessment. Therefore, it is not possible to say whether and how the different visualisation methods possibly affected the interpretation of the data.

In the result section (line 217), the authors state that *“From the 69 comparisons, 37 (54%) fulfilled the criterion for expert consensus”*, without explaining which type of consensus is considered here. In the methods section, two types of consensus are considered.

Figure 2: although the ordering of the bars within each row offers a visually gradient from left to right, it prohibits interpreted of a set of opinions regarding one and the same trial (baseline, follow-up, drug-efficacy) as these are in different positions in each of the three rows. I would therefore suggest to only order the bars in the first row (baseline), and then maintain the same order of questions in the second and third row.

I do not understand the statement *“If only those comparisons with expert consensus are considered, the agreement was generally slightly higher but the overall picture did not change”* (lines 253-237). Higher than what? And what picture? I’m assuming the authors mean to refer to a preceding more general statement, but not clear which one it is (e.g. that the arithmetic mean performed worst?).

In the caption to figure 3, it is unclear what is meant by *“N (from left to right): 22, 22, 23, 7, 14, 16”* in the last sentence. Are these the number of expert opinions on which the raw percentage agreements were based (which wouldn’t be many)? It would help if the authors explained why these numbers are lower than the number of opinions that were elicited. I am finding myself again and again trawling back to the methods section to try to interpret the results. It is explained somewhere, but I am at the point where I am giving up the effort to try and understand.

Further, it took me quite some effort to understand (I hope I do) that the two columns in figure 3 relate to the two ways to assess the expert opinions (“simple majority” vs. “absolute majority”). However, the terms used in the results section and the figure (“all” vs. “consensus”) in no way refer back to the terminology used in the methods section (i.e. “simple majority” vs. “absolute majority”). Please harmonise and simplify.

Then last regarding the figure 3 caption; *“The rank denotes the rounded mean rank calculated separately for each column.”*. I assume it should say “row” instead of “column” as these are ranks in terms of performance of the different mean metrics, correct? Also, it’s not clear what the rank is based on: “all” or “consensus” and “baseline”, “follow-up”, and/or “drug efficacy”. Please clarify.

Figure 4 caption: very technical description and difficult to wrap the mind around: *“Scores that favored arm B were converted [abs (score – 6)]. In this case the colors of the associated bar plots and the sign of the difference were reversed, too.”* So, if I understand correctly, the authors are basically saying that all results are expressed in favour of whichever trial arm showed a higher drug efficacy, and in there was no clear difference, in favour of arm A.

Although I can appreciate the cheerful confetti colours of Figure 4 (and the joy the maker must have experienced creating it – I do love *ggplot2* myself as well), I am honestly overwhelmed by everything going on in this figure and have no clue how to interpret it. After reading the main text, it would help considerably if the authors spaced the different panels, add markers indicating “baseline”, “follow-up”, and “drug-efficacy”, and provide some guidance in the figure 4 caption on what the different panels describe (e.g. highlighting that the fourth panel is some detailed info of the three data points form the second panel). I would further suggest to consider to drop the loess curve (or at least its SE) because it is just wild, rather distracting, and is itself not even discussed in the results section (and doesn’t have to be, for that matter).

In the result section under sensitivity analysis, please state the number of trials that was excluded in the sensitivity analysis (i.e. three; I had to count the bars in figure 2 and the analogous plot in suppl. S2).

Lines 331-332: *“Although we found the best performance with a parameter of 0.2, a higher parameter of 0.4 might be more practical.”* I don’t follow. Please clarify why this would be more practical.

Lines 332-334: *“Likewise, the Lehmer means require a constant in the presence of zero values and despite it performed similar to the Hölder mean, we would not recommend its use.”* Why?

Some general thoughts on the relevance and implications of this study:

- The problem is – first and foremost – the small sample sizes used in some drug efficacy trials. The ensuing noise can be “dealt with” by using “robust” summary metrics, at a cost of bias (e.g. the geometric mean bias) and or statistical precision (e.g. truncating reduces sample size and increases statistical uncertainty). Still, the arithmetic mean is the most unbiased, and it is straightforward to express uncertainty about it in both descriptive stats and regression models.
- In their analysis, the authors do not consider (or at least discuss) measures of uncertainty but only present point-estimates for egg counts at baseline and follow-up and the estimated drug efficacy.
- I am relieved that the authors at least say that the arithmetic mean shouldn’t be banned, because we need it for meta-analyses. As the authors say, arithmetic means work best in large, population-based studies. Wouldn’t this metric then also be best for meta-analyses? Also, the proposed alternative methods are not (yet) amenable with meta-analysis and would be incompatible with existing literature.
- The authors highlight limitations of previous studies as: *“Several researchers have continued to identify the most appropriate method for calculating egg reduction rates using empirical data or computer simulations. However, the methods used were based on assumptions about true efficacy or egg distribution, which favored one specific mean over another [17,18,20,21].”* This needs more explaining (it’s a two-sentence paragraph) as it comes across as a gross generality. “Knowing” the truth (even if fictive) would generally be considered desirable, even if only for the purpose of the experiment. The authors could would have further addressed this by including some simulated data in the questionnaire to the experts, and seeing how well the experts’ interpretations/opinions matched the “true” process that generated the fictive data. This would also have provided a pseudo-benchmark for how well experts can interpret data like this.

Minor textual revisions:

1) Line 80: “The ERR is defined as [**the]** relative reduction…”.
2) Line 101: first use of the abbreviation “MCT” without explanation. I’m guessing its “mean calculation technique” or “metric of central tendency”…
3) Lin3 181: “Moreover” is used, but there is no argument being made here. “Further”, “next”, “also” etc. is appropriate here.
