# Supplementary material for: One mean to rule them all? The arithmetic mean based egg reduction rate can be misleading when estimating anthelminthic drug efficacy in clinical trials
Source: PLoS Negl Trop Dis. 2020 Apr 8;14(4):e0008185. doi: 10.1371/journal.pntd.0008185 (PMC7170292; doi:10.1371/journal.pntd.0008185)
Supplement: Attachment — Submitted filename: PNTD-D-19-01393-Point_by_point_response_v1.docx [file pntd.0008185.s005.docx]

#PNTD-D-19-01393 **(Moser *et al.*) – Point-by-Point response**

Title: One mean to rule them all? The arithmetic mean based egg reduction rate can be misleading when estimating anthelminthic drug efficacy in clinical trials

Dear Sara Lustigman, dear Matthew C Freeman,

We were delighted to receive the encouraging feedback from the editors and reviewers regarding our manuscript. In the meantime, we have made an effort to revise our manuscript in light of the reviewers’ comments, and feel that the overall quality of our manuscript has been further improved. Below, please find our point-by-point response to the editors and reviewers, clearly indicating how and where in the manuscript changes have been made (all line numbers refer to the revised manuscript). To assist you in readily tracking our amendments, we have highlighted all of our changes made in the revised manuscript with a yellow marker. We also enclose a “clean” version of the revised manuscript.

We very much hope that this revised version of our manuscript and is suitable for publication in *PLOS Neglected Tropical Diseases*, and hence, we look forward to your response.

Yours sincerely,

Jan Hattendorf (on behalf of all authors)

*****************************************

Reviewer #1

1.1 The authors present an interesting and (as far as I can see) novel approach towards selection between summary measures of central tendancy in calculating egg reduction rates. The rationale is clear, the methods relating to expert elicitation appear to be sound, and the paper is mostly well written. However I do have some concerns relating to the way in which the "agreement" between summary statistics and expert assessments is discussed and presented that I would like the authors to address before publication:

We thank the reviewer for this encouraging statement and are confident that we adequately addressed all points in the revised manuscript.

1.2 The paper is currently lacking a clear discussion of the fundamental difference between different types of mean at the population level, and the difference between the population mean and sample mean. When reading this paper I think the reader could get the impression that there is in reality "one true mean (to rule them all!)" but of course this is not the case; the population arithmetic mean and the population geometric mean are fundamentally different for any skewed distribution, and it is to be expected that the arithmetic mean of a sample is the best estimator of the population arithmetic mean, and similarly that the geometric mean of a sample is the best estimator of the population geometric mean. I suspect that the authors understand this well enough (from e.g. their discussion of the Dobson et al article) but I think they could do more to spell it out for the less statistically-experienced reader.

We absolutely agree with the reviewer. Only one important detail we have to correct: we put a question mark not an exclamation mark behind the statement “One mean to rule them all?” Anyway, the point raised by the reviewer is important and valid. Because the topic is related to the next but one point raised by the reviewer, we ask to read our response to point 1.4.

1.3 What is interesting and useful about this paper is that they make no a-priori assumption regarding which population mean is most relevant: they simply present the distributions to experts and ask which shows the best "average" (in a general sense) egg reduction. I therefore read the results more as an ellicitation exercise where the experts are tasked with selecting which of the population means best reflects their interpretation of the data; and we find that the geometric mean best reflects their qualitative feeling about the distributions. This is most likely a result of the experts tending to down-weight outliers in a similar way to the geometric mean, which is interesting. I wonder how much of this is related to the potential issue regarding refusal of treatment on lines 302-304: do the authors think that the experts were potentially dismissing extreme values as non-representative due to the potential for this type of effect? I think there is scope for a lot more discussion in this area - although it will naturally stray into social science issues with which I am not very familiar, so it could well be that some of these issues are already well described elsewhere.

Certainly, some people might consider extreme values as non-representative and ignore them but we are not sure if this the most likely explanation for all experts because other reasons are also plausible. A statistician might consider a drug which cures one-half of the participants and has no effect on the other half similarly effective compared to a drug reduces all egg counts by 50% but a public health specialist (or even a transmission dynamics modeller) might have another view. Other people might have primarily the health burden aspect in mind and prefer a majority of persons with only a light infections, which are for many species asymptomatic. Most people would likely prefer to suffer 1 week from a heavy disease than 10 weeks from a mild disease (depending on the disease of course). We also have to keep in mind that a reduction of eggs does not necessarily mean the same reduction in worms, because the egg per worm output is in some species density dependent. Although a discussion of this point is very speculative, we agree with the reviewer that the reader is currently left a bit alone with results and discuss the point briefly in the revised version (lines 335-339).

1.4 However, I do think the authors are somewhat over-playing their conclusions - it is not possible based on their data to conclude that the geometric mean is in any sense a "better" measure than the arithmetic mean, rather that the experts they asked tended to down-weight outliers in a similar way to the geometric mean. This is an important point in itself - face validity is a desirable property of such a statistic - but it is not sufficient to recommend that the geometric mean be favoured in all situations. For example, the arithmetic mean remains the best indicator of the total number of eggs being excreted, and therefore the epidemiological infection pressure within the environment. I also think it is meaningless to use sample geometric means when comparing egg reduction rates to another population where arithmetic means were used: this suggests that future studies should present both arithmetic mean reduction for comparison with previous studies as well as geometric mean reduction (or some other metric) to give an estimate that may correspond more closely to an expert evaluation of the distributions. As the paper stands I think these issues could easily be misinterpreted, so I do feel that this needs to be addressed.

We absolutely agree with the reviewer. It was not our intention to extrapolate our results beyond human drug trials. There are many areas where i) an arithmetic mean is for biological reasons more plausible, e.g. environmental contamination as the reviewer correctly pointed out, ii) “outliers” or of particular interest, e.g. super shedders in Schistosomas elimination settings, iii) an individual instead of a population egg reduction measure is required, e.g. if main focus is on treatment failures or in resistance surveillance, iv) outliers don’t play a big role because of sample size as in large scale program monitoring, v) etc …

We don’t believe that we promote the geometric mean as the universally better estimate, e.g.: in the title we explicitly refer to the clinical trial setting, in the abstract we recommend that the arithmetic mean should be supplemented by the geometric mean in drug trials, in the authors summary we refer to “anthelmintic human drug trials, which are characterized by small sample size” and in the discussion we “our results should not be extrapolated to large scale program evaluations.”. To avoid any over-interpretation we included a new statement in the introduction (lines 106-108) and extended this point in the discussion at a more prominent position (lines 317-318) and we discuss this point again at the end of the discussion together with point 1.2 raised by the reviewer in (lines 375-380).

1.5 I am not sure I accept your argument that raw agreement is appropriate - what is to be lost by calculating a Kappa statistic? This is a very standard method that naturally incorporates the chance agreement probability, and also allows you another way of calculating 95% confidence intervals. Please either do this or give a better justification for not doing so.

We calculated the weighted Kappa (or more precisely the ICC which is approximated by Kappa) only to assess the inter-rater reliability among the experts. To compare the expert opinion with the calculated measures of central tendency Kappa is not very useful because it represents the observed agreement with respect to a baseline agreement defined as “the agreement that would be expected due to random allocation, given the quantities specified by the marginal totals of the contingency table”. Therefore, it depends on the prevalence. A simple example shows why this is not relevant in our context:

Let us consider the following 2 examples:

|  |  | Calculated | mean |  |
| --- | --- | --- | --- | --- |
|  |  | A better | B better |  |
| Expert | A better | 25 | 2 |  |
| opinion | B better | 2 | 25 | Kappa = 0.85 |

|  |  | Calculated | mean |  |
| --- | --- | --- | --- | --- |
|  |  | A better | B better |  |
| Expert | A better | 50 | 2 |  |
| opinion | B better | 2 | 0 | Kappa = -0.04 |

Kappa is 0.85 in the first example but -0.04 in the second. Because A and B have no real meaning in our context (the trial arm pairs are randomly assigned to A or B) the tables are equivalent and should result in the same estimate.

1.6 Line 49: I know what you mean by "problematic in skewed distributions" but I would prefer you to describe the issue (i.e. sensitive to extreme values) directly, as it is not necessarily a "problem" in a statistical sense if it yields the best unbiased estimator of the population arithmetic mean (which it typically does)

It is not only question of statistical properties; a mean should also represent the data well. Many statistical textbooks state that the arithmetic mean is not appropriate to summarise heavily skewed data some authors speak even from misuse (note that in this context the focus is on summarizing data not on drawing conclusions). However, we agree that the sensitivity to extreme values is likely the most important aspect – especially in our context. We revised the article (line 50):

The arithmetic mean is problematic in skewed distributions mainly because the mean is sensitive to outliers, whereas…

1.7 Line 74: I am not sure that "While" is appropriate to start this sentence - it seems like you mean something more like resistance is widespread in animal parasites and therefore is also likely in humans? Perhaps rephrase.

We rephrased as suggested.

1.8 Line 197: Should be "Data from six publications"

We corrected the sentence as suggested

1.9 Lne 321-322: It seems like this could use a reference

We added a reference (Dobson 2009)

1.10 I found appendix S1 very useful - thanks for including it. If I were to be critical, I would suggest that density plots are not a particularly useful way of representing these data as they 'smooth out' outliers - histograms (with an appropriate bin) or even empirical cumulative distribution plots may have been better. But the B+W plots are fine so I don't think it's a big problem.

In retrospect, we would also prefer histograms, because they are more easy to interpret. We were concerned that automatically generated histograms could be misleading if breaks and bin widths are not appropriately chosen. There is unfortunately nothing we can do.

Reviewer #2

2.1 Moser and colleagues assessed the agreement in interpreting drug efficacy data applying a variety of measures for central tendency. Based on the differences in agreement across the experts they conclude that the current recommended arithmetic mean should be reported together with an estimate which is more robust to outliers, e.g. geometric mean. Overall, I have three major concerns on the paper for which I would like to see an in depth response.

We are confident that we have addressed the points satisfactory.

2.2 First, one should not only interpret the efficacy of drugs based on a measure of central tendency only. Instead this should be interpret along the uncertainty intervals around the point estimate. I therefore strongly believe that the recommendation should not focus on including different measures of central tendency (which often give totally different point estimates), rather we need to recommend reporting the appropriate 95% confidence intervals. This is a well established practice in veterinary parasitology, but not in the human parasitology (see also WHO guidelines were conclusions on drug efficacy are based solely based on point estimates).

We apologize that one could get the impression that we would recommend to present point estimates without their corresponding measures of uncertainty. We assumed it goes without mentioning that reporting of a point estimates implies reporting of the corresponding interval estimate as well. The reviewer will not find any study of any of the co-authors where the estimate of the primary outcome is reported without its confidence or credible intervals. Even in this manuscript, we report confidence intervals for the raw agreements, which was an extremely challenging exercise. We now state explicitly in the conclusion that point estimates should always be reported together with the corresponding confidence intervals (lines 386-387). On the other hand, point and interval estimates are two completely different things and one cannot use one for the purpose of the other. Please read our response to 2.4, where we further elaborate on this point using a concreate example.

2.3 Second, I strongly doubt whether the applied methodology is appropriate. To me this exercise can only be assessed through a simulation study where a substantial number of datasets with varying mean and skewness of egg counts, number of outliers, sample size and true underlying drug efficacy are generated. Subsequently, the different measures of tendency are applied and there deviation from the true underlying efficacy is then assessed. This would indeed allow for more evidence-based recommendations.

We agree with the reviewer in the fact that simulations represent an extremely important and powerful tool. But we believe that simulations are strong in supporting decisions but decisions should not be based on simulations alone. One problem in this case is that simulations are based on assumptions and that these assumptions will favour one measure. Let us take up the idea of the reviewer and assume a simple simulation to estimate drug efficacy of a single drug. Egg counts are modelled as negative binomial distribution and egg reduction as some kind of relative reduction. Because the expected value of a negative binomial distribution is the arithmetic mean the use of this mean will provide appropriate and efficient point and interval estimates and will outperform all other means (given appropriate statistical techniques are used). In another simulation we change our assumptions and include different probabilities of treatment failures. If we play with skewness and sample size we will easily find combinations where the geometric mean will perform better. Please see also our response to comment 3.21where we provide examples from the literature. As mentioned before, we believe that simulations are useful and important but without external input they are difficult to interpret. We respect the view of the reviewer but we believe that our study makes a contribution. As the reviewer points out in 2.9 one contribution could be to guide the design and interpretation of future simulations.

2.4 Third, the authors often make references to the field of veterinary parasitology. Although there are indeed clear similarities between the field of veterinary and human parasitology, there are some important differences which, in my opinion, are not always accurately reflected in the current draft. For example, cure rate is not used at all in veterinary parasitology to assess the efficacy of anthelmintic drugs. In contrast to WHO, who only recently recommended ERR based on the arithmetic mean, its veterinary counter part (World Association for the Advancement of Veterinary Parasitology; WAAVP), has been recommending reporting both arithmetic mean and 95% CI since 1992. I therefore would like to propose that the authors either explicitly mention these differences in to the manuscript, or re-direct the focus of the manuscript to the human field only. I would prefer the latter given the focus of the journal, the dataset used and the link to WHO guidelines.

In line with the other reviewers the reviewer hat the same feeling that the reader could get the impression that we would like to extrapolate our results beyond human drug trials. As we already explained before, that was not our intention. In the revised manuscript, we point this out at even more and more prominent positions (please see our response to 1.4). With respect to the confidence intervals: the point estimate (i.e. the mean) and the interval estimate (i.e. the confidence interval) are two different things which are used for two different purposes. The reviewer cites the WAAVP guidelines for the detection of anthelmintic resistance (Coley 1992). The guideline says that if the lower bound of the 95% CI of the egg reduction rate is below 90% (and the point estimate above 95%) resistance should be suspected. This is in fact a hypothesis test and therefore the CI is of course appropriate. If the 95% CI includes 90% we cannot accept the hypothesis “We are confident that the egg reduction rate is at least 90%” and the presence of resistance cannot be ruled out. If we look at the WAVVP guidelines on evaluating anthelminthic drug efficacy from the same time period (Woods et al 1999, Veterinary Parasitology 58, 181-213) the following formula is recommended to estimate drug efficacy in a placebo controlled trial:

(geometric mean control group – geo. mean treatment group) / geo. mean control group

i.e. only point estimates are used. Of course, if we would like to know if the reduction is statistically significant different from 0 we need to calculate the confidence intervals (which was at that time likely not available for this problem).

Besides the clarifications that the results should not be intrperted beyond human drug trials (see also our response to 1.4), we also note in the revised document explicitly that the results should not be extrapolated to the veterinarian sector (line 312 and line 318). With respect to the WHO guidelines: several scientists from the veterinarian sector were part of the writing committee. We would also like to challenge the view of the reviewer that there is a broad a consensus in the veterinarian sector: Coles (1992), Wood (1995), Smothers (1999), Rozsa (2000), Neuhauser (2004), and Dobson (2009) provide a set of contradictory advice on how to calculate egg reduction.

2.5 Line 67: Abbreviate soil-transmitted helminths as STHs instead of STH

Thanks for spotting this error.

2.6 Line 77: I tend to disagree that recommendation for anthelmintic drugs the primary output would be different from a clinical medicine and public health; this is in particular when drugs are not 100% and CR is affected by the analytic sensitivity of the diagnostic tool. In addition, in veterinary parasitology, CR is not recommended at all, and hence this sentence may need some revision (see also general comment). Note that reference 4 are not the WAAVP guidelines, Coles et al., 1992 Vet Parasitol are.

We agree with the 2^nd^ point of the reviewer (CR are usually not used in vet science) and reworded the sentence accordingly (line 80). However, clinical trials (humans) have usually CR as primary outcome while the WHO recommends ERR to monitor large scale interventions. The reviewer is right: ref 14 is not the guidelines but this is on purpose.

2.7 Line 85: I would strongly recommend not only draw conclusions on the measure of tendency only; taken the uncertainty intervals on board would be quite essential to draw conclusions. It is therefore my belief, that this statement deviates from the fundamental approach to interpret point estimates.

See our response to 2.1

2.8 Line 88: it will be important to highlight that each of these different measures of central tendency result in quite different point estimates, which will only further create confusion. Again, one should not only focus on the point estimate.

We cite in the manuscript a trial conducted by Speich et al. with a bit more than 100 participants in each arm. The A+I arm showed a cure rate of 30% and an ERR (based on the arithmetic mean) of 85.6%, whereas the A+O arm showed a cure rate of 74% and a ERR of 72.5%. We strongly believe that complementing the arithmetic mean with the geometric mean has decreased the confusion not increased.

2.9 Line 95: it is not clear to me why assumptions on true drug efficacy or egg distributions would favour one specific mean over the other one. In fact, I strongly believe that this is the only way forward (see also general comment). However, I tend to agree that the simulation studies did not include outliers, that is why it would be good to include this in newly designed simulation study.

See or response to point 2.3

2.10 Line 123: I found the presentation of the egg counts (providing boxplots separately for baseline and follow-up) rather misleading. Now the experts only have some idea on the central tendency, but not on the individual variation in response across treatment arms (are high excretes at baseline still excreting most or the least eggs at follow-up). As a consequence of this are not provided with all the information to make a proper on judgement on the difference in drug efficacy across treatment arms. This might have been done on purpose, but it once more highlights the need for interpreting the point estimate with uncertainty intervals.

Yes, paired boxplots connected with lines would have been a good idea because the sample size is only moderate. We will consider this point in the future.

2.11 Line 303: I found this sentence rather redundant, it is not really relevant here, or I mis understood the message. Do you mean that arithmetic can be used to assess infection intensity but not for drug efficacy or is a difference made between trials to designed to assess / compare drugs and trials designed to assess efficacy of drugs in large scale deworming progams?

With ‘large scale program evaluations’ we tried to refer to both: cluster randomized trials and intervention/program monitoring. In both scenarios the impact of outliers is smaller. In cluster randomised trials outliers are more likely to be balanced simply because the sample size is usually above 1000 per arm.

2.12 Line 305: in general ERR would not really recommended at all to assess differences in drug efficacy in dose-response trials or in any other trial designed to assess differences. This is because the variation in point-estimates might be to large, that is why CR is probably better (given that subjects are randomized treatment arms stratifying for baseline egg counts).

The reviewer is right that the ERR would rarely be used as primary outcome in human drug trials but it is commonly used as secondary outcome. The question which mean should be preferred is not limited to the primary outcome.

2.13 Line 310: I am not sure whether this statement holds true; reporting ERR based on group means does not exclude variation due to individual response, as long as the uncertainty intervals around the point estimate are provided. As a consequence of this, the following argumentation on the use of simple measures of central tendency over complex models does not hold true anymore (see also first major comment).

This is a quotation and not our own argument. However, we found that the word “require” was not adequately reflecting the original statement. We replaced the word with “which can be more precisely assessed with” (line 321).

2.14 Line 348 – 353: overall I largely disagree with the drawn conclusions. The authors are providing the wrong message. In stead of providing 2 different measures of central tendency, which only creates confusion, one should emphasize the need for reporting uncertainty intervals, which may already reflect the impact of outliers.

We agree that this is not an optimal and hopefully not a long term solution. The current guidelines are recommending the arithmetic mean and we cannot simply say “Stop reporting it!” (and in other situations the arithmetic mean is adequate). However, the mean was in so strong disagreement with the expert opinion and showed no correlation at all with the cure rates that we also cannot recommend to continue with the current way. With respect to the uncertainty interval, we have in this case an opposite view: a point estimate can either be appropriate or not. The confidence interval does not change the point estimate at all, i.e. an inappropriate point estimate is not getting more appropriate because it is complemented with its confidence intervals.

Reviewer #3

3.1 Overall the manuscript is well-written, although inconsistencies in use of terminology and potential lack of detail (possibly as a result of the former) make it difficult to interpret the results in several instances. As a result, I was finding myself trawling back and forth between methods and results section to try and understand what was done and why and what it means. This doesn’t make for a very pleasant reading experience (2 hours from top to bottom of the paper) and is a distraction from the work’s content. Below, I provide some pointers on how to potentially improve this.

We are aware the fact that the paper is not always easy to follow. The approach is new (at least as far as we, and reviewer 1, can see) and has no established terminology, we have different types of agreement (agreements among raters, agreement between raters and means and in the appendix agreement among means), besides the different types of means we have calculated we have the mean score, … . In many cycles we exchanged terms to make the content more clear and digestible, obviously with varying success. Therefore, we are extremely grateful for the careful revision, which will improve the manuscript not only content-wise but also, improves the readability.

3.2 Supplement S1 (example questionnaire): it is very useful that the authors include this example. I’ve tried to assess a few of the trial arms, and realised how very challenging it actually is. Before having looked at the results, I was expecting a lot of variation between the experts’ answers for some of the comparisons. Was the first page all the information that the invited experts got? Or was there additional information? I am mentioning this because: 1) It is not clear to me what “graph 1-3” refers to in “The upper row (graph 1-3) shows eggs per gram.”. Should it say “graph 1-2”? I only see two panels (baseline and follow-up) in the top left box with the egg counts on the natural scale. It would be clearest if the panels are marked with a number of capital letter, and that these are used for reference. 2) “The box-plots present the min, Q1, median, Q3, max, dots represent outliers.” This seems inaccurate/incomplete; the ends of the whiskers on the box-plots cannot represent the minimum and maximum as there are also “outliers”. Most likely, if these box-plots where generated in R with ggplot2 under the default settings, the whiskers represent at most the maximum, but no further than some constant times the IQR. And outliers are then defined as any data points outside this maximally allowed range of the whiskers. This needs to be clarified. Then again, maybe this is how it was presented to the experts… 3) What is the base of the logarithm being used for transformation of egg counts? Base 10 or is it the natural logarithm (or some other value)? The values on the y-axis of the logarithmic plots are uninterpretable this way. I am guessing the natural logarithm was used, based on looking at an outlier around 3000 epg that translated to a value of about 8 on the “log scale” (e^8 ~= 3000).

Besides a short welcome mail, the questionnaire was the only information the participants got. With respect to clarity we can only say i) we never got requests for qualification by mail or by phone, ii) we achieved a response rate higher than expected (roughly two third) and iii) there were very few missing data. Anecdotal feedback of exports within our own institution showed that some found it easy and other challenging to fill out but main criticism was that it was too long. The reviewer is right that the reference to figures 1 to 3 in the upper row is a bit confusing. In an earlier draft of the questionnaire we numbered the example figures from 1 to 6 (the density plots were considered as 2 different graphs because they are in separate boxes). The boxplots were not generated with ggplot2 but with basic stats package with default options (outliers are considered as any point more than 1.5 times box-length times away from the box margins) and the reviewer is absolutely right that the whiskers do not present the min/max if outliers are present so strictly speaking: “the lowest data point excluding any outliers”. However, we trust that all participants were familiar with boxplots – we were more concerned about the density plots. We also agree with the reviewer that it would be more precise to add that the base of the logarithm is e. Of note, a different base will not change the shape or the number or position of outliers as the example with 100 uniform rand numbers in the picture below shows. It will only change – as the reviewer correctly points out – only the values on the y axis.

There is not much we can do now; we thank the reviewer for the detailed feedback, which will improve future studies. Besides that, we included in the manuscript the information that we used R’s stats package with default settings. The revised manuscript reads now (lines 131-133):

The plots were separately generated for baseline and follow-up and were represented on linear and log scale using R’s stats package with default settings (except for the smoothing bandwidth of the density plots which was set as the maximum egg counts of both trial arms divided by 20).

3.3 Although I am reasonably versed in applied stats and math, it is not clear to me what property is described by the equation (5) on line 160. Is M(.) the quantile function used, and does this mean that the result it returns will be the same if the input is entered in duplicate?

Yes, the mean should be the same if the same input is duplicated. Precisely: Given an n-tuple e of n elements, the mean should satisfy M(e) = M({e, e}), or in easy words: the mean of 1,2,4,8 should be the same as the mean of 1,1,2,2,4,4,8,8. Although, that might sound trivial for many readers this is not the case for most quantile functions. We thought that our formulation would be easier to understand for the majority of readers but this seems to be not the case. In our revision, it is now technically correct (lines 167-168).

3.4 The argument to only apply truncation or winsorization to the right tail (high values) is not convincing. Egg counts of zero can be just as “extreme” in a sample where most egg counts are much higher. It is an inherent property of the negative binomial distribution of egg counts; both zeroes and high values can be considered extreme.

The reviewer is right. There, might be settings where also zero or low egg counts represent extreme values. We assume that this will be rather rarely the case in clinical trials (although a person in a control arm was accidentally treated or was treated outside the trial). However, as can be seen in the questionnaire, this was not the case in any of the trials used for this study. Anyway, the reviewer raised a fair point we took up in the discussion of the revised manuscript (lines 365-367).

3.5 From the methods section (lines 184-185), it is not clear to me how the “raw percentage agreement” between the experts’ opinions and each of the mean metrics was calculated. What was considered agreement? In the preceding paragraph (lines 168-182), it is explained how the expert opinion was summarised over the whole group (in two ways), but I can’t tell whether or how these summaries where used for the calculation of the raw percentage agreement. Expert opinions were scored on a 5-point Likert scale; were the quantitative mean metrics and the differences between arms somehow translated to the 5-point Likert scale and then compared to the experts’ opinions? Or was the Likert scale dichotomised (if so, how?) and then compared to presence of a statistically significant difference in the quantitative mean metrics (ignoring the magnitude of a potential difference)? This needs a more explaining.

We revised the paragraph and are confident that it is now easier to understand (lines 176-189). Please see also our response to the related point 3.7. In brief: we dichotomised both variables into “more eggs in A” and “more eggs in B” (or “higher egg reduction in A/B”). Expert opinion was converted into the same categories but with 2 different definitions (to ensure that the results are robust in this respect): ‘all studies’ (simple majority): more experts say arm A has more/slightly more eggs than experts say B has more/slightly more eggs (with a somewhat difficult algorithm to break ties). ‘consensus studies’ (absolute majority): more experts says A has more/slightly more eggs than undecided experts and those with a opposite view together, i.e. studies where not the overwhelming number of reviewers shared the same view have been excluded.

3.6 Although innovative and exciting (I would have definitely enjoyed to participate in the questionnaire), a weakness of the methodology used in this paper is that there is no way to benchmark how well the experts’ review of the trial result actually was, as the “true” differences between trials arms and differences in drug efficacy were not known. It may well be that our visual interpretation of data like these is inherently biased. The authors tried to address this by offering different visualisation methods, but it was up to each expert reviewer to choose what visualisation method to use primarily for their assessment. Therefore, it is not possible to say whether and how the different visualisation methods possibly affected the interpretation of the data.

What the reviewer express as a weakness we consider as one of the central features of the study. Of course, if the visual interpretation is strongly biased, we would not have a serious limitation but the whole study would be pointless. There is no way that we can proof the opposite but we have some indications that this might not the case: i) although it was up to each expert to choose the preferred visualisation method, it is likely that some experts used primarily the boxplots, others the density plots and the remaining both but nevertheless there is a high agreement among experts, ii) the experts judgement correlated very well with cure rates. We think that it is at least plausible that the experts made a fair assessment. If the raters agreement with a mathematical measure of central tendency is not much more than flipping a coin, we argue that something is strange and should be sorted out before the next version of guidelines will be published.

3.7 In the result section (line 217), the authors state that “From the 69 comparisons, 37 (54%) fulfilled the criterion for expert consensus”, without explaining which type of consensus is considered here. In the methods section, two types of consensus are considered.

We apologize that this terminology was a bit hidden in the previous version. We agree that this makes thing complicated. In simplified the revised manuscript and we repeat the definition of “experts consensus” at first occurrence in the result section and in the figure captions.

3.8 Figure 2: although the ordering of the bars within each row offers a visually gradient from left to right, it prohibits interpreted of a set of opinions regarding one and the same trial (baseline, follow-up, drug-efficacy) as these are in different positions in each of the three rows. I would therefore suggest to only order the bars in the first row (baseline), and then maintain the same order of questions in the second and third row.

Very good idea. Although, we modified the suggestion a bit and ordered it according to the 3^rd^ row (efficacy).

3.9 I do not understand the statement “If only those comparisons with expert consensus are considered, the agreement was generally slightly higher but the overall picture did not change” (lines 253-237). Higher than what? And what picture? I’m assuming the authors mean to refer to a preceding more general statement, but not clear which one it is (e.g. that the arithmetic mean performed worst?).

The sentence becomes clear if it gets clearer that it refers to the right column of figure 3. We agree with the reviewer that the term ‘picture’ was not a good choice and replaced it with pattern. We revised the manuscript accordingly (line 246-248).

3.10 In the caption to figure 3, it is unclear what is meant by “N (from left to right): 22, 22, 23, 7, 14, 16” in the last sentence. Are these the number of expert opinions on which the raw percentage agreements were based (which wouldn’t be many)? It would help if the authors explained why these numbers are lower than the number of opinions that were elicited. I am finding myself again and again trawling back to the methods section to try to interpret the results. It is explained somewhere, but I am at the point where I am giving up the effort to try and understand.

We apologize for being imprecise. N is not the number of trial arm pairs to be evaluated by both, the experts and the calculated means. We revised the figure caption which nor reads:

Raw percentage agreement between expert opinion and the calculated means for egg burden are at baseline and follow-up egg and drug efficacy (superiority of a certain trial arm). Both, expert opinion and calculated means were dichotomized into 'A > B' and 'B > A'. Number of trial arm comparisons N: left panel: bl=22, fu=22, ef=23; right panel; bl=7, fu=14, ef=16. AM: arithmetic mean, GM: geometric mean, Hö: Hölder mean, Le: Lehmer mean, Wi: winsorized mean, tr: truncated mean. Numbers behind Hö/Le indicate parameter p, numbers behind Wi/tr denote proportion discarded/replaced. The rank denotes the rounded row mean rank. All: simple majority definition, consensus: absolute majority criterion, more than 50% of experts favor one arm, i.e. only those comparisons marked with footnote a) in Fig 2 are considered.

3.11 Further, it took me quite some effort to understand (I hope I do) that the two columns in figure 3 relate to the two ways to assess the expert opinions (“simple majority” vs. “absolute majority”). However, the terms used in the results section and the figure (“all” vs. “consensus”) in no way refer back to the terminology used in the methods section (i.e. “simple majority” vs. “absolute majority”). Please harmonise and simplify.

Please see our response to 3.7.

3.12 Then last regarding the figure 3 caption; “The rank denotes the rounded mean rank calculated separately for each column.”. I assume it should say “row” instead of “column” as these are ranks in terms of performance of the different mean metrics, correct? Also, it’s not clear what the rank is based on: “all” or “consensus” and “baseline”, “follow-up”, and/or “drug efficacy”. Please clarify.

We were incorrect. We used the term column instead of panel/heatmap. We revised the figure legend (see response to 3.10).

3.13 Figure 4 caption: very technical description and difficult to wrap the mind around: “Scores that favored arm B were converted [abs (score – 6)]. In this case the colors of the associated bar plots and the sign of the difference were reversed, too.” So, if I understand correctly, the authors are basically saying that all results are expressed in favour of whichever trial arm showed a higher drug efficacy, and in there was no clear difference, in favour of arm A.

We agree that the description was short and confusing. We revised the figure capture.

**Fig 4. Relationship between the calculated difference among 2 trial arms estimated by different means and experts' rating scores** The symbols in the first 3 panels show the association between the rater scores and the differences in egg counts or egg reductions between trial arm pairs calculated by 4 different means (different means are represented by different colors). The lines represent the corresponding loess smoothing lines. The bar plots at the top show the experts’ ratings in the same way as in Fig 2. Some bar plots were placed at the bottom to avoid over plotting. Note, that rater scores (and bar plots) which favored arm B have been converted to favor arm A, e.g. a rating score of 4 would be converted to a score of 2 (a score of 3 indicates no difference between the trial arms). In 3 comparisons at follow up (numbered 1 to 3 in the top right panel) the estimates were especially strong diverging. The corresponding raw data are presented as strip plot and histogram in the bottom right panel.

3.14 Although I can appreciate the cheerful confetti colours of Figure 4 (and the joy the maker must have experienced creating it – I do love ggplot2 myself as well), I am honestly overwhelmed by everything going on in this figure and have no clue how to interpret it. After reading the main text, it would help considerably if the authors spaced the different panels, add markers indicating “baseline”, “follow-up”, and “drug-efficacy”, and provide some guidance in the figure 4 caption on what the different panels describe (e.g. highlighting that the fourth panel is some detailed info of the three data points form the second panel). I would further suggest to consider to drop the loess curve (or at least its SE) because it is just wild, rather distracting, and is itself not even discussed in the results section (and doesn’t have to be, for that matter).

ggplot2? All figures in the manuscript are generated with R’s basic plotting functions with more than 200 lines of code to just generate figure 4! We modified the figure as suggested by the reviewer and agree that it looks now tidier. We kept the loess curve but dropped the confidence bands.

3.15 In the result section under sensitivity analysis, please state the number of trials that was excluded in the sensitivity analysis (i.e. three; I had to count the bars in figure 2 and the analogous plot in suppl. S2).

It is written in the first sentence of the paragraph.

3.16 Lines 331-332: “Although we found the best performance with a parameter of 0.2, a higher parameter of 0.4 might be more practical.” I don’t follow. Please clarify why this would be more practical.

The sentence should be read together with the previous sentence. In the revised manuscript we combined both into a single sentence to be clearer (lines 353-356)

Considering the above stated example with 9 times 0 egg counts and one time 1000 eggs, the geometric mean would estimate a mean egg count of 1, whereas the Hölder mean (with parameter 0.2) would estimate 0.01; therefore, a higher parameter of 0.4 might be more appropriate.

3.17 Lines 332-334: “Likewise, the Lehmer means require a constant in the presence of zero values and despite it performed similar to the Hölder mean, we would not recommend its use.” Why?

The geometric mean has been criticized that the estimate depends on the constant to be added and that the choice of the constant is arbitrary (although nowadays almost always a 1 is used).

3.18 Some general thoughts on the relevance and implications of this study: The problem is – first and foremost – the small sample sizes used in some drug efficacy trials. The ensuing noise can be “dealt with” by using “robust” summary metrics, at a cost of bias (e.g. the geometric mean bias) and or statistical precision (e.g. truncating reduces sample size and increases statistical uncertainty). Still, the arithmetic mean is the most unbiased, and it is straightforward to express uncertainty about it in both descriptive stats and regression models.

We completely agree with the reviewer that the main problem in drug trials is the limited number of participants. If the number of participants would be larger the problem of outliers would disappear or at least smaller. We also agree with the reviewer that the arithmetic mean has many nice statistical properties and that many statistical procedures are based on the arithmetic mean. We included this point in our discussion (lines 371-374).

3.19 In their analysis, the authors do not consider (or at least discuss) measures of uncertainty but only present point-estimates for egg counts at baseline and follow-up and the estimated drug efficacy.

The reviewer is right, we did not assessed the performance of the different means in terms of power to detect a significant difference. Although, this would have been different it was beyond the scope of this study. We agree that it is a point neglected in the discussion, it is only very briefly discussed in the last paragraph (the last but one paragraph in the revised version). We could only speculate about power but we included a statement on available statistical tools. See our response to the previous point.

3.20 I am relieved that the authors at least say that the arithmetic mean shouldn’t be banned, because we need it for meta-analyses. As the authors say, arithmetic means work best in large, population-based studies. Wouldn’t this metric then also be best for meta-analyses? Also, the proposed alternative methods are not (yet) amenable with meta-analysis and would be incompatible with existing literature.

Fair point. We included this suggestion in our discussion (line 373).

3.21 The authors highlight limitations of previous studies as: “Several researchers have continued to identify the most appropriate method for calculating egg reduction rates using empirical data or computer simulations. However, the methods used were based on assumptions about true efficacy or egg distribution, which favored one specific mean over another [17,18,20,21].” This needs more explaining (it’s a two-sentence paragraph) as it comes across as a gross generality. “Knowing” the truth (even if fictive) would generally be considered desirable, even if only for the purpose of the experiment. The authors could would have further addressed this by including some simulated data in the questionnaire to the experts, and seeing how well the experts’ interpretations/opinions matched the “true” process that generated the fictive data. This would also have provided a pseudo-benchmark for how well experts can interpret data like this.

If we define the “true” efficacy is the %-reduction of eggs and we use negative binomial distributed data with any parameters mu and shape. Next we have a drug which ‘kills’ each egg with a probability P. Do we really need a simulation to proof that the arithmetic mean will outperform all other in estimating P? If we define performance as Smoothers did in 1999: … (2) influence of extreme values, and (3) proximity to the median. Wouldn’t it be enough to remember our first undergraduate stats lecture to know that the geometric mean will perform better in skewed distributions? If we follow Montresor 2007 and we focus on morbidity with a definition of efficacy as the proportion of individuals changing from moderate/high egg category to the low egg category. Is it worth to hypothesis unrealistic scenarios to show that sometimes the arithmetic and sometimes the geometric mean yielding inappropriate results? (Please see also our responses to comments 1.3, 2.3 and 3.6.) It is easy to explain it in examples but difficult to describe it in a concise way but we followed the recommendation of the reviewer and extended the paragraph and provided examples in the revised manuscript (lines 96-100). With respect to the inclusion of simulated data in the questionnaire we would like to cite reviewer #1 “What is interesting and useful about this paper is that they make no a-priori assumption regarding which population mean is most relevant”. The “truth” in this study is the opinion of the experts. Let us reconsider our example from response 1.3: we include some simulated data for two drugs with identical baseline distributions. Drug A reduces the egg counts of all participants by 50% while Drug B cured half of the participant and the egg counts of the other half do not change at all. Our “true” simulated reduction is an “arithmetic mean” reduction and both drugs are similarly efficacious from an arithmetic means perspective. Afterwards one expert says “In my opinion the drugs are not similar” can we respond “You are wrong because we simulated the data”?

3.22 Line 80: “The ERR is defined as [the] relative reduction…”.

Modified as suggested definition

3.23 Line 101: first use of the abbreviation “MCT” without explanation. I’m guessing its “mean calculation technique” or “metric of central tendency”…

The reviewer is almost right: “measure of central tendency” we do not use the abbreviation anymore in the revised manuscript.

3.24 Lin3 181: “Moreover” is used, but there is no argument being made here. “Further”, “next”, “also” etc. is appropriate here.

We replaced moreover with “further” as suggested.
