# Supplementary material for: One mean to rule them all? The arithmetic mean based egg reduction rate can be misleading when estimating anthelminthic drug efficacy in clinical trials
Source: PLoS Negl Trop Dis. 2020 Apr 8;14(4):e0008185. doi: 10.1371/journal.pntd.0008185 (PMC7170292; doi:10.1371/journal.pntd.0008185)
Supplement: Attachment — Submitted filename: PNTD-D-19-01393_R1 LC.docx [file pntd.0008185.s006.docx]

General comments

Congratulations to the authors for producing a revised manuscript that is much improved in clarity of both text and figures. It has helped me better understand what was done, has reassured me of the rigour of the study and the analysis, and now makes for a pleasant reading experience. Only a few minor points need further clarification (details below). Other than that, I endorse acceptance for publication.

Methods section

In line 176-178, the authors explain *“To assess agreement between experts and calculated means we dichotomized both variables. For the calculated means we simply used the difference between both arms to decide of arm A or arm B showed higher egg counts or egg reductions, ignoring the magnitude of the difference.”* What about the statistical significance of the difference? Was any difference considered a “difference” when comparing analysis results to the experts’ opinions?

The use of the word “studies” in the definitions “all studies” and “consensus studies” is still somewhat confusing. As I understand it, these two definitions are used to summarise the experts’ opinion about drug efficacy in a particular study (i.e. not a summary over multiple studies). The definitions in brackets (simple vs. absolute majority criterion) make more sense. I therefore suggest that the authors only use the latter terminology.

Lines 191-192: *“Further, we explored the relationship between the calculated differences among the trial arm pairs and the difference of the observed CRs.”* Do the authors mean the relationship between calculated ERRs and observed CRs?

Lines 194-195: *“Performance was calculated as the raw percentage agreement between experts and each mean.”* What are the numerator and denominator of this percentage? I can guess: the number of concordant scores for all pairs of expert opinion and observed differenced (i.e. summed over all data sources and experts) divided by the total number of scores (number of experts times number of data sources). Or was concordance first summarised per study (or per expert) and then average over all experts (or studies)? This should be clarified for the sake of reproducibility.

Lines 199-200: *“In addition, we calculated Krippendorff's alpha for ordinal metrics.”* What is this a measure of? Level of corcordance of expert scores and observed differences? Or of inter-rater reliability (something that is mentioned in the preceding sentence). If those things are the same, then I am afraid I might be misunderstanding something.

Lines 227-229: *“From the 69 comparisons, 37 (54%) fulfilled the criterion for expert consensus (i.e. absolute majority criterion - more than 50% of experts favor one arm).”* Again, I suggest using only the terminology “absolute majority criterion”, seeing that the term “consensus studies” (as mentioned in methods) is not used in the results section. Further, I was expecting to also read here what proportion of the 69 comparisons fulfilled the simple majority criterion (to see whether or not it is very close to the 54% for the absolute majority criterion).

Caption to Figure 2: the authors should indicate which rows of stacked bars within the figure correspond to the expert scores for differences in 1) baseline egg counts, 2) follow egg counts, and 3) drug efficacy. I assume the order represent aforementioned metrics in that order from top to bottom, but I can’t be sure from the caption or the figure itself.

Figure 3: I suggest to use “simple majority” and “absolute majority” in the headers above the two columns (instead of “all” and “consensus”).

Lines 276-278: *“Consequently, the arithmetic mean performed better in the sensitivity analysis, where small trials were excluded (S2 File, Fig S3) but showed still the poorest performance among the four investigated means.”* Figure S3 in supplement S2 looks exactly the same as Figure 4 in the main manuscript. If I look at the LOESS lines for arithmetic mean, I don’t see a difference between Figure S3 and Figure 4 even though the main text suggests an improvement. Is it possible that the wrong figure was pasted into the supplement? Or if they are not identical, could you perhaps indicate in Figure 4 which of the studies were based on a sample size ≤30 (e.g. with an open circle around the cross symbol) so the reader can understand which studies were excluded in the supplement?

Minor textual revisions:

- Line 90: “…homogeneity of the variance between the compared group**s**…”
- Line 143: remove dash in “…fill-in…”.
- Lines 344-345: “Another issue represents the fact that the mean is not defined for **samples that include** zeros.”
- Lines 362-363: “For obvious reasons, the estimate is not suitable for high CRs, since all CRs above 90% would result in an estimate of ~~0~~ **100% CR[?]**.”
