# Supplementary material for: One mean to rule them all? The arithmetic mean based egg reduction rate can be misleading when estimating anthelminthic drug efficacy in clinical trials
Source: PLoS Negl Trop Dis. 2020 Apr 8;14(4):e0008185. doi: 10.1371/journal.pntd.0008185 (PMC7170292; doi:10.1371/journal.pntd.0008185)
Supplement: Attachment — Submitted filename: PNTD-D-19-01393-Point_by_point_response_R2.docx [file pntd.0008185.s007.docx]

#PNTD-D-19-01393 **(Moser *et al.*) – Point-by-Point response**

Title: One mean to rule them all? The arithmetic mean based egg reduction rate can be misleading when estimating anthelminthic drug efficacy in clinical trials

Dear Sara Lustigman, dear Matthew C Freeman,

We are deeply impressed that the reviewer took the time to read the manuscript with so much care a 2^nd^ time. We revised the manuscript as suggested and added a new supplementary appendix with a detailed example explaining the reader agreements, scores and how figures are related to each other. Below, please find our point-by-point response, indicating how the comments where addressed. We have highlighted all of our changes made in the revised manuscript with a yellow marker. We also enclose a “clean” version of the revised manuscript.

We very much hope that this revised version of our manuscript and is suitable for publication in *PLOS Neglected Tropical Diseases*, and hence, we look forward to your response.

Yours sincerely,

Jan Hattendorf (on behalf of all authors)

*****************************************

Reviewer #1

Thanks for considering my suggestions - I think your additions have greatly improved the manuscript, and it is now much clearer from reading the main paper what exactly you mean by "performance".

I have only a couple of further comments in relation to the abstract (which I think is currently identical to the previous version): Lines 34-36 currently states: "Among all investigated means, the arithmetic mean showed poorest performance and agreed with the expert opinion in only 64% (bootstrap confidence interval: 60−68)." I think this is still misleading as it suggests that it BOTH showed poorest performance AND agreed with expert opinion less than the others, whereas in fact you judge performance based on expert opinion. How about something like: "Among all investigated means, the arithmetic mean showed the poorest performance with only 64% agreement with expert opinion (bootstrap confidence interval: 60−68).". Line 39: I think the comma should be between 'CI: 78-87)' and 'but' rather than between 'well' and 'after'. Line 42: The wording of "necessarily provide reliable estimates in anthelminthic efficacy" is also a bit vague and potentially misleading. How about something like: "necessarily rank anthelminthic efficacies in the same order as might be obtained from expert evaluation of the same data". Line 60: Could you make it more explicit exactly what you mean by performance here, eg: "...showed the poorest performance in terms of agreement with expert opinion"

We incorporated all comments exactly as suggested.

Reviewer #3

Congratulations to the authors for producing a revised manuscript that is much improved in clarity of both text and figures. It has helped me better understand what was done, has reassured me of the rigour of the study and the analysis, and now makes for a pleasant reading experience. Only a few minor points need further clarification (details below). Other than that, I endorse acceptance for publication.

We thank the reviewer for this encouraging statement.

Methods section: In line 176-178, the authors explain “To assess agreement between experts and calculated means we dichotomized both variables. For the calculated means we simply used the difference between both arms to decide of arm A or arm B showed higher egg counts or egg reductions, ignoring the magnitude of the difference.” What about the statistical significance of the difference? Was any difference considered a “difference” when comparing analysis results to the experts’ opinions?

The reviewer raised 2 points here: i) difference between egg counts in trial arm pairs and ii) difference in the 'performance' of the means (defined as agreement). For i) we did not consider significance for mainly practical reasons: as far as we now there are no standard formulas to calculate confidence intervals for e.g. differences in Winsorized or Hölder means. With respect to ii) the study was not sufficiently powered to detect significant differences among the raw agreements. We discussed this issue at design stage but we concluded that we simply do not have enough studies, besides that the length of the questionnaire was already at its limit. The only thing we did is to construct bootstrap confidence intervals of the mean agreement but also those should be interpreted with care because we resampled expert but not studies.

The use of the word “studies” in the definitions “all studies” and “consensus studies” is still somewhat confusing. As I understand it, these two definitions are used to summarise the experts’ opinion about drug efficacy in a particular study (i.e. not a summary over multiple studies). The definitions in brackets (simple vs. absolute majority criterion) make more sense. I therefore suggest that the authors only use the latter terminology.

The reviewer is right that the simple & absolute majority criterion is related to a single study. But later on we select all studies where the simple or the absolute criterion is fulfilled. We now have a new supplementary appendix (S2 File, the former S2 is now S3) which gives clear guidance on: how agreement and scores are calculated, how simple & absolute majority arw related to all & consensus studies and how the different figures relate to each other.

Lines 191-192: “Further, we explored the relationship between the calculated differences among the trial arm pairs and the difference of the observed CRs.” Do the authors mean the relationship between calculated ERRs and observed CRs?

Thanks for spotting this error. We refer here to figure 5, where we compare cure rates with both: the difference in ERR and the scores.

Lines 194-195: “Performance was calculated as the raw percentage agreement between experts and each mean.” What are the numerator and denominator of this percentage? I can guess: the number of concordant scores for all pairs of expert opinion and observed differenced (i.e. summed over all data sources and experts) divided by the total number of scores (number of experts times number of data sources). Or was concordance first summarised per study (or per expert) and then average over all experts (or studies)? This should be clarified for the sake of reproducibility.

The new appendix is clarifying everything.

Lines 199-200: “In addition, we calculated Krippendorff's alpha for ordinal metrics.” What is this a measure of? Level of corcordance of expert scores and observed differences? Or of inter-rater reliability (something that is mentioned in the preceding sentence). If those things are the same, then I am afraid I might be misunderstanding something.

We agree with the reviewer that the statement is not clear. The revised sentence reads: "Inter-rater reliability among experts' judgment was assed by Krippendorff's alpha for ordinal metrics. We also estimated the intra cluster …"

Lines 227-229: “From the 69 comparisons, 37 (54%) fulfilled the criterion for expert consensus (i.e. absolute majority criterion - more than 50% of experts favor one arm).” Again, I suggest using only the terminology “absolute majority criterion”, seeing that the term “consensus studies” (as mentioned in methods) is not used in the results section. Further, I was expecting to also read here what proportion of the 69 comparisons fulfilled the simple majority criterion (to see whether or not it is very close to the 54% for the absolute majority criterion).

We followed the suggestion of the reviewer. The sentence in the revised manuscript reads: " From the 69 comparisons, 37 (54%) fulfilled the absolute majority criterion, i.e. more than 50% of experts favor one arm and 67 (97%) fulfilled the simple majority criterion."

Caption to Figure 2: the authors should indicate which rows of stacked bars within the figure correspond to the expert scores for differences in 1) baseline egg counts, 2) follow egg counts, and 3) drug efficacy. I assume the order represent aforementioned metrics in that order from top to bottom, but I can’t be sure from the caption or the figure itself.

It is shown in the figure but easy to overlook. We added the information also in the figure caption.

Figure 3: I suggest to use “simple majority” and “absolute majority” in the headers above the two columns (instead of “all” and “consensus”).

We tried to modify the figure as suggested but the words were too long. We left the figure unchanged but think that it should not a problem anymore, because we have the new supplementary appendix.

Lines 276-278: “Consequently, the arithmetic mean performed better in the sensitivity analysis, where small trials were excluded (S2 File, Fig S3) but showed still the poorest performance among the four investigated means.” Figure S3 in supplement S2 looks exactly the same as Figure 4 in the main manuscript. If I look at the LOESS lines for arithmetic mean, I don’t see a difference between Figure S3 and Figure 4 even though the main text suggests an improvement. Is it possible that the wrong figure was pasted into the supplement? Or if they are not identical, could you perhaps indicate in Figure 4 which of the studies were based on a sample size ≤30 (e.g. with an open circle around the cross symbol) so the reader can understand which studies were excluded in the supplement?

Thanks for spotting this mistake. We corrected the figure in the revised appendix.

Minor textual revisions:

-	Line 90: “…homogeneity of the variance between the compared groups…”

-	Line 143: remove dash in “…fill-in…”.

-	Lines 344-345: “Another issue represents the fact that the mean is not defined for samples that include zeros.”

-	Lines 362-363: “For obvious reasons, the estimate is not suitable for high CRs, since all CRs above 90% would result in an estimate of 0 100% CR[?].”

We incorporated all comments exactly as suggested.
